# Prediction of additive genetic variances of descendants for complex families based on Mendelian sampling variances

**DOI:** 10.1093/g3journal/jkae205

**Published:** 2024-08-28

**Authors:** Tobias A M Niehoff, Jan ten Napel, Mario P L Calus

**Affiliations:** Animal Breeding and Genomics, Wageningen University & Research, Droevendaalsesteeg 1, P.O. Box 338, 6700 AH Wageningen, The Netherlands; Animal Breeding and Genomics, Wageningen University & Research, Droevendaalsesteeg 1, P.O. Box 338, 6700 AH Wageningen, The Netherlands; Animal Breeding and Genomics, Wageningen University & Research, Droevendaalsesteeg 1, P.O. Box 338, 6700 AH Wageningen, The Netherlands

**Keywords:** Mendelian sampling variance, Mendelian sampling, usefulness criterion, progeny variance, within-family selection, multiparental crosses, Index5, genomic selection, response to selection, genetic variance

## Abstract

The ability to predict the outcome of selection and mating decisions enables breeders to make strategically better selection decisions. To improve genetic progress, those individuals need to be selected whose offspring can be expected to show high genetic variance next to high breeding values. Previously published approaches enable to predict the variance of descendants of 2 future generations for up to 4 founding haplotypes, or 2 outbred individuals, based on phased genotypes, allele effects, and recombination frequencies. The purpose of this study was to develop a general approach for the analytical calculation of the genetic variance in any future generation. The core development is an equation for the prediction of the variance of double haploid lines, under the assumption of no selection and negligible drift, stemming from an arbitrary number of founder haplotypes. This double haploid variance can be decomposed into gametic Mendelian sampling variances (MSVs) of ancestors of the double haploid lines allowing usage for non-double haploid genotypes that enables application in animal breeding programs as well as in plant breeding programs. Together with the breeding values of the founders, the gametic MSV may be used in new selection criteria. We present our idea of such a criterion that describes the genetic level of selected individuals in 4 generations. Since breeding programs do select, the assumption made for predicting variances is clearly violated, which decreases the accuracy of predicted gametic MSV caused by changes in allele frequency and linkage disequilibrium. Despite violating the assumption, we found high predictive correlations of our criterion to the true genetic level that was obtained by means of simulation for the “corn” and “cattle” genome models tested in this study (0.90 and 0.97). In practice, the genotype phases, genetic map, and allele effects all need to be estimated meaning inaccuracies in their estimation will lead to inaccurate variance prediction. Investigation of variance prediction accuracy when input parameters are estimated was not part of this study.

## Introduction

Traditionally, breeders select the best individuals as parents of the next generation, be it based on phenotypes or based on estimated breeding values (BVs). This method of truncation selection aims to maximize the genetic gain in the short term. If genetic gain is to be maximized in the mid or long term, the genetic diversity of selected individuals, as well as the compatibility of potential mates, should also be considered.

To maximize the response to selection in the long-term, optimum contribution selection (OCS) has been developed (Meuwissen 1997). When using OCS, the BV is no longer the sole criterion based on which selection decisions are made, because OCS also considers the relationships among selection candidates. Typically, OCS aims to reduce the rate of increase of average coancestry, measured as the increase in identity-by-descent relationships among all individuals born per generation. This, in turn, aims to minimize the loss of diversity at loci relative to founders or some base generation (Woolliams *et al.* 2015; Meuwissen *et al.* 2020).

While OCS does limit the rate of inbreeding, it does not directly maximize genetic variance of a particular trait. If we consider the genetic variance of progeny of a mating as a selection criterion in addition to the parent average BV, the expected future genetic variance is explicitly considered for selection decisions. Under the infinitesimal model, the gametic Mendelian sampling variance (MSV), which is needed to derive the progeny variance, follows from the inbreeding level of individuals (Dempfle 1990). However, this is just an expectation and the actual MSVs differ beyond the effect of homozygosity alone, due to linkage and a finite number of loci in the genome (Hill and Weir 2011). For illustration, correlations between the estimated gametic MSV of various traits and genomic inbreeding levels in dairy cattle have been reported to be indeed negative, but very weak, ranging from −0.03 to −0.23 (Segelke *et al.* 2014; Santos *et al.* 2019).

Several criteria have been developed in animal breeding research that aim to select matings or animals in such a manner that the fraction of their offspring showing a BV higher than a certain selection threshold is maximized (Santos *et al.* 2019; Wellmann and Bennewitz 2019; Bijma *et al.* 2020). A similar criterion for the selection of promising crosses known as the “usefulness criterion” has been proposed in plant breeding research by Schnell and Utz (1975). According to its original definition, the usefulness criterion describes the expected BV of the best inbred line that can be selected among a defined number of sister inbred lines obtained from a cross of inbred lines. All these criteria have in common that they do not only consider the BV of individuals, or the average BV of parents, but also the expected distribution of BVs of progeny of a mating or a cross. In Niehoff *et al.* (2024), we recently proposed a new criterion (ExpBVSelGrOff) that aims to maximize the BVs of grandoffspring of a particular mating. For this criterion, not only the distribution of BVs of the offspring, as considered in all proposed criteria before, but also the distribution of BVs of the grandoffspring are considered. The key component that enabled this development was the prediction of the gametic MSV of selected offspring that we assumed have the same gametic MSV as random offspring. Our proof-of-concept study (Niehoff *et al.* 2024), which used simulations, showed that considering an additional future generation in the selection criterion results in higher genetic gain than the usefulness criterion (Schnell and Utz 1975) or Index5 (Bijma *et al.* 2020) without compromising diversity.

The distribution of BVs for polygenic traits of progeny of a mating is typically assumed to follow a normal distribution that can be described by a mean and a variance. The expected average BV of offspring is equal to the parent average BV. The variance in the offspring generation can be obtained by simulating offspring in silico (Segelke *et al.* 2014; Mohammadi *et al.* 2015) or with analytical approaches developed in plant breeding research (Lehermeier *et al.* 2017; Osthushenrich *et al.* 2017; Allier, Moreau, *et al.* 2019; Wolfe *et al.* 2021) and in animal breeding research (Bonk *et al.* 2016; Santos *et al.* 2019; de Abreu Santos *et al.* 2020; Musa and Reinsch 2023). Both in silico simulations and analytical approaches require the same information as input, namely phased genotype information of potential parents of a mating, marker effects, and recombination frequencies between markers.

Generally, the focus of approaches in animal breeding is to predict the variance of BVs of gametes produced by an animal. The approaches developed in plant breeding focus on predicting the variance of lines produced from a cross of 2 fully inbred founder lines, either obtained after several rounds of selfing or by creating double haploids (DHs). A significant extension to calculate genetic variances for more complex crosses in which up to 4 different haplotypes, i.e. 4 different fully inbred parents, may be involved, was proposed by Allier, Moreau, *et al.* (2019). In Niehoff *et al.* (2024), we showed how the variance of DH lines derived from 4 fully inbred founder lines can be used to derive the average gametic MSV of offspring of a mating of 2 outbred individuals that enables planning 2 generations ahead in an animal breeding setting. We speculated that new selection criteria can be devised that look even more generations ahead if there were methods to predict the gametic MSV of a further future generation of descendants. Thus far, no other approaches have been presented that enable planning more than 2 generations ahead, i.e. considering more than 4 founding haplotypes.

In this paper, we present a method to calculate the variance of DH lines derived from an arbitrary number of fully inbred founder lines analytically, building upon the approach by Allier, Moreau, *et al.* (2019), and expectations under the infinitesimal model. Equations to convert these DH variances into gametic MSV are also presented, and the equations are validated by comparison to variances of MSV computed from simulated descendants. These gametic MSVs enable further development of new criteria that look even more generations ahead. For livestock breeding, such a criterion could look like the criterion presented in Equation (1). This criterion aims to predict the expected average genetic level, or BV when ignoring non-additive effects, of selected individuals in the F4 generation (Expected Breeding Value of Selected Great-Great-Grand-Offspring). These are great-great-grandoffspring to their founders that need to be selected in the current generation. This criterion can be understood as adding up the selection differential that is expected in future generations. In addition to the BVs of the founders, this criterion requires knowledge of the genetic variation within families in each future generation that is being considered. We introduce this criterion here to explain why we are interested in predicting genetic variation in future generations. The details of this criterion are discussed further in the *Discussion* section, as the main focus of this study is on developing equations to predict future genetic variances.


(1) $$\begin{array}{rl} & ExpBVSelGrGrGrOff\\ & =\;\frac{BV_{AB}+BV_{CD}+BV_{EF}+BV_{GH}+BV_{IJ}+BV_{KL}+BV_{MN}+BV_{OP}}{8}+\\ & i_{F1}\left(\frac{\sigma_{F1\;ABxCD}+\sigma_{F1\;EFxGH}+\sigma_{F1\;IJxKL}+\sigma_{F1\;MNxOP}}{4}\right)+\\ & i_{F2}\left(\frac{\sigma_{F1\;ABCDxEFGH}+\sigma_{F1\;IJKLxMNOP}}{2}\right)+\\ & i_{F3}\sigma_{F1\;ABCDEFGHxIJKLMNOP}+\\ & i_{F4}\sqrt{2\sigma_{gametes\;F1\;ABCDEFGHxIJKLMNOP}^{2}} \end{array}$$

In practice, the accuracy of variances predicted with our presented method depends on the accuracy of estimated phase states, marker effects, and recombination frequencies. The prediction accuracy will differ between breeding programs, e.g. because of differences in sizes of their reference populations used for genomic prediction, which is why those effects were not included in this study.

## Methods

In this section, we first review the quantitative genetic model that explains how genetic variances are passed from one generation to the next (*Review of variance transmission*). Secondly, we review the prediction of DH variances as presented by Allier, Moreau, *et al.* (2019) as this is crucial for the developments presented in this manuscript (*Review of Allier, Moreau, et al. (2019)*). The third subsection starts by describing how the genetic variance in a future generation can be decomposed into gametic MSVs of their ancestors (*Quantitative genetic motivation*). This is followed by a detailed description of the key development of this manuscript, namely the prediction of variances among descendants of an arbitrary future generation by considering QTL effects and haplotypes of the founders (*Connection between infinitesimal model and haplotype-based approach*). Then, we show how these variances can be converted to gametic MSV (*Conversion to gametic MSV*). We end by describing the simulation setup we used to validate our equations (*Validation with simulation*).

### Review of variance transmission

This section reviews the quantitative genetic model that is commonly used for prediction of variances of BVs of the next generation. The genetic variance of the next generation of a population can be modeled as shown in Equation (2) that is based on equation 16.21b of Walsh and Lynch (2018) and extended to include the covariance between Mendelian sampling terms passed on by the sire and the dam ($COV(gamMS_{Sires_{t}},gamMS_{Dams_{t}})$).


(2) $$\sigma_{A_{t+1}}^{2}=\frac{\sigma_{A_{Sires_{t}}}^{2}}{4}+\frac{\sigma_{A_{Dams_{t}}}^{2}}{4}+\frac{COV(A_{Sires_{t}},A_{Dams_{t}})}{2}+\sigma_{gamMS_{Sires_{t}}}^{2}+\sigma_{gamMS_{Dams_{t}}}^{2}+2COV(gamMS_{Sires_{t}},gamMS_{Dams_{t}})$$

where $\sigma_{A_{Sires_{t}}}^{2}$ and $\sigma_{A_{Dams_{t}}}^{2}$ describe the genetic variances of the groups of sires and dams at time *t*, $COV(A_{Sires_{t}},A_{Dams_{t}})$ describes the covariance of BVs of the mated sires and dams, $\sigma_{gamMS_{Sires_{t}}}^{2}$ and $\sigma_{gamMS_{Dams_{t}}}^{2}$ describe the gametic MSVs of the sires and dams at time *t*, and $\sigma_{A_{t+1}}^{2}$ is the genetic variance in the next generation. $COV(gamMS_{Sires_{t}},gamMS_{Dams_{t}})$ is the covariance of the gametic Mendelian sampling terms of the sire and the dam. It describes how well a gamete with high BV produced by the sire predicts the BV of the gamete produced by the dam. Since Mendelian sampling events are independent under natural conditions, this term is usually assumed to be null. One notable exception is the case of DH lines as explained further below. The sum of the terms $\frac{\sigma_{A_{Sires_{t}}}^{2}}{4}$, $\frac{\sigma_{A_{Dams_{t}}}^{2}}{4}$, and $\frac{COV(A_{Sires_{t}},A_{Dams_{t}})}{2}$ describes the variance of parent average BVs. Under random mating, $COV(A_{Sires_{t}},A_{Dams_{t}})$ is expected to be null as the BV of a sire has a correlation of 0 to the BV of the dam. Under assortative mating based on performance, i.e. better males are on average mated to better females, this covariance term is positive. Under disassortative mating, this covariance term is negative.


(3) $$\sigma_{A_{t+1}}^{2}=\sigma_{A_{Individuals_{t}}}^{2}+2\sigma_{gamMS_{Individuals_{t}}}^{2}$$

In the special case that individuals are selfed like in some plant breeding scenarios, Equation (2) can be rewritten as shown in Equation (3). This is equivalent to the theoretical case of animals producing full-sib offspring with themselves. The intuitive reason for why $\sigma_{A_{Individuals_{t}}}^{2}$ does not need to be divided by anything is that the variance of parent averages is identical to the variance of BVs of individuals. With selfing, the sire and dam variances are identical and the correlation between sire and dam BV is 1. Together, this results in the covariance being identical to the variance of BV of the sire and dam.


(4) $$\sigma_{A_{t+1}}^{2}=\sigma_{A_{Individuals_{t}}}^{2}+4\sigma_{gamMS_{Individuals_{t}}}^{2}$$

In the even more special case that DH progeny is generated from the individuals of the population, Equation (2) changes to Equation (4). The difference between offspring obtained from selfing to offspring obtained by inducing doubling of haploid genomes is that the Mendelian sampling events are independent when selfing whereas they are not when DH lines are produced. A DH is produced by doubling the set of chromosomes of a gamete, implying that the gametic Mendelian sampling deviation from the sire's BV is the same as the deviation from the dam's BV. The correlation between the gametic MS deviation of sire and dam is 1, and since the gametic MSV of sire and dam is identical, the covariance of the MS deviations ($COV(gamMS_{Sires_{t}},gamMS_{Dams_{t}})$) is identical to the gametic MSV. This also shows that the additive genetic variance of DH lines produced from a single F1 plant is 4 times the additive gametic MSV of that F1 plant. More directly, it can be shown that the variance among DHs is always 4 times as large as the variance among gametes that would be used to produce the DHs as follows: $Var(BV_{DH})=Var(2BV_{gamete})=2^{2}Var(BV_{gamete})=4Var(BV_{gamete})$ (ploidy-level squared). A small intuitive example based on haplotypes and recombination for this phenomenon is provided in the Appendix.

**Fig. 1.**
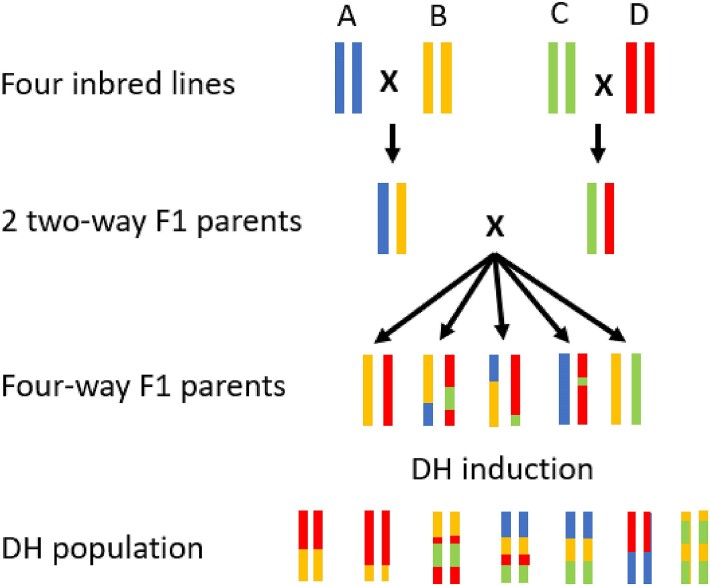
Example for chromosome transmittance to DH lines as in the 4-way cross considered by Allier, Moreau, *et al*. (2019). The figure is modified from Niehoff *et al*. (2024).

Allier, Moreau, *et al.* (2019) presented an analytical approach to calculate the variance of DH lines derived from the cross of 4 fully inbred founder lines. The cross in question is shown in Fig. 1. Assuming the 4 fully inbred lines are unrelated, one can view a 2-way F1 parent as shown in Fig. 1 as a non-inbred animal. Thus, the variance of DH lines derived from a 4-way cross can be decomposed into the gametic MSV of 4-way F1 parents and the variance of BVs of these 4-way F1 parents. Under the infinitesimal model, the gametic MSV of individuals ($\sigma_{gamMS_{Sires_{t}}}^{2}$ and $\sigma_{gamMS_{Dams_{t}}}^{2}$ in Equation (1)) may be predicted based on the individual's inbreeding level as $\sigma_{gamMS_{individual}}^{2}=0.25*(1-F_{individual})*\sigma_{A}^{2}$, with $\sigma_{A}^{2}$ as the genetic variance in the random mating non-inbred base population (Dempfle 1990). Thus, we derive that the expected variance of BVs of 4-way F1 individuals is $0.5\sigma_{A}^{2}$ (Equation (5)).


(5) $$\begin{array}{rl} \sigma_{4-way\;F1}^{2} & =\frac{\sigma_{A_{\;first\;2-way\;F1}}^{2}}{4}+\frac{\sigma_{A_{second\;2-way\;F1}}^{2}}{4}+\sigma_{gamMS_{\;first\;2-way\;F1}}^{2}+\sigma_{gamMS_{second\;2-way\;F1}}^{2}\\ & =\frac{0}{4}+\frac{0}{4}+\frac{\sigma_{A}^{2}}{4}+\frac{\sigma_{A}^{2}}{4}\\ & =0.5\sigma_{A}^{2} \end{array}$$

This variance, when combined with the gametic MSV of the 4-way F1 parents and considering that the variance of DH lines produced by a single individual is always 4 times larger than its gametic MSV, allows us to derive that the variance of BVs among DH lines produced from a 4-way cross of unrelated founders is expected to be 1.5 times as large as the additive genetic variance in the base population (Equation (6)).


(6) $$\begin{array}{rl} \sigma_{4-way\;DH}^{2} & =\sigma_{4-way\;F1}^{2}+{\bar{\sigma }}_{gamMS_{4-way\;F1}}^{2}*4\\ & =0.5\sigma_{A}^{2}+\frac{\sigma_{A}^{2}}{4}*4\\ & =1.5\sigma_{A}^{2} \end{array}$$

Thus, the variance of 4-way F1 parents, who are identical to full-sib offspring in an animal breeding setting, together with the variance of DH lines produced by the 4 inbred founders can be used to derive the average gametic MSV of a random 4-way F1 parent as shown by Niehoff *et al.* (2024) as (Equation (7)):


(7) $${\bar{\sigma }}_{gamMS_{4-way\;F1}}^{2}=\frac{\left(\sigma_{4-way\;DH}^{2}-\sigma_{4-way\;F1}^{2}\right)}{4}$$

### Review of Allier, Moreau, *et al.* (2019)

This section reviews and summarizes the key development of Allier, Moreau, *et al.* (2019) that is relevant for this study. The focus is on the equations for calculating the variance of a DH family derived from 2 and 4 fully inbred founder lines without any intermediate step of selfing. The discussed equations are presented and shown in Table 1 of this study, and Supplementary File S1 of Allier, Moreau, *et al.* (2019). R code to calculate these variances is provided in Supplementary File S3 of the follow-up study by Allier, Lehermeier, *et al.* (2019). Allier, Moreau, *et al.* (2019) presented a method to calculate the genotypic covariance ($\Sigma_{\;jl}$) in progeny between two loci *j* and *l* based on the disequilibrium parameter ($D_{\;jl}$) in pairs of parental lines and the frequency of recombination between the loci ($c_{\;jl}^{\left(1\right)}$) in a meiotic event (see Table 1 of Allier, Moreau, *et al.* 2019). The element $c_{\;jl}^{\left(1\right)}$ can be calculated as follows:


$$c_{\;jl}^{\left(1\right)}=\frac{1-e^{-2d_{\;jl}}}{2}$$

where $d_{\;jl}$ is the distance in Morgan units between the loci *j* and *l*. If both loci are not on the same chromosome, $c_{\;jl}^{\left(1\right)}$ is 0.5 because the two loci are not linked. For this conversion equation from Morgan units to recombination frequencies, Haldane's mapping function is assumed here (Haldane 1919), but any other mapping function can be used too. If a matrix Σ is constructed with the dimensions *pxp* with *p* QTL in the genome and every element of matrix Σ corresponding to $\Sigma_{\;jl}$, the progeny variance can be calculated as $\sigma_{Progeny}^{2}=\beta^{\prime }\;\Sigma \beta$, where β is a *p* dimensional vector containing QTL effects. The interpretation of matrix element $\Sigma_{\;jl}$ is that it describes the linkage disequilibrium (LD) between loci *j* and *l* in the progeny.

**Table 1.** Matrix element $\Sigma_{\;jl}$ for QTLs *j* and *l* for DH progeny, depending on the cross design.

	$\Sigma_{\;jl}$
2-way	$4(1-2c_{\;jl}^{\left(1\right)})D_{\;jl}^{AB}$
4-way	$\left(1-2c_{\;jl}^{\left(1\right)}{)}^{2}(D_{\;jl}^{AB}+D_{\;jl}^{CD})+(1-2c_{\;jl}^{\left(1\right)})(D_{\;jl}^{AC}+D_{\;jl}^{AD}+D_{\;jl}^{BC}+D_{\;jl}^{BD}\right)$

The calculation of matrix element $\Sigma_{\;jl}$ differs per type of cross considered. Here, the most relevant types to consider are DH lines produced by 2-way and a 4-way cross. For these types, $\Sigma_{\;jl}$ can be calculated according to Table 2 S1 in Supplementary Material S1 of Allier, Moreau, *et al.* (2019) and as shown in Table 1 here.

$D_{\;jl}^{AC}\;$ , $D_{\;jl}^{AD}$, $D_{\;jl}^{BC}$, $D_{\;jl}^{BD}$, $D_{\;jl}^{AB}$, and $D_{\;jl}^{CD}$ are the linkage disequilibria between pairs of loci in the parental haplotypes. These are 0 in case at least one locus shows the same allele in both haplotypes, −0.25 in case the 2 haplotypes are in repulsion phase, and 0.25 in case the haplotypes are in coupling phase. Also, if the indicators *j* and *l* refer to the same locus and the 2 haplotypes carry different alleles at this locus, meaning they would produce a heterozygous F1 individual if crossed, then the disequilibrium parameter is 0.25. The values for $D_{\;jl}$ for all possible phase states for two loci are shown in Table 2.

**Table 2.** Value of linkage disequilibrium parameter $D_{\;jl}$ for different phase states when comparing 2 haplotypes.

Type	Phase$\left(H_{1_{j}}-H_{1_{l}}\;/\;H_{2_{j}}-H_{2_{l}}\right)$	$D_{jl}$
Double heterozygous	1-1/0-0	0.25
	1-0/0-1	−0.25
Single homozygous	0-1/0-0	0
	1-0/0-0	0
	0-1/1-1	0
	1-0/1-1	0
Double homozygous	1-1/1-1	0
	0-0/0-0	0
	1-0/1-0	0
	0-1/0-1	0

The genome in this example has only two loci. The linkage disequilibrium parameter *D* here refers to the linkage between loci *j* and *l* where j≠l. $H_{1_{j}}$ is an indicator for the allele on haplotype 1 at locus *j*, and $H_{1_{l}}$is an indicator for the allele on haplotype 1 at locus *l* and so forth.

### Extension to arbitrary number of founder haplotypes

In the previous subsections, we described the general quantitative genetic model for variance transmission and the method of Allier, Moreau, *et al.* (2019) that enabled us to predict the variance of DH lines based on up to 4 founder haplotypes, recombination frequencies, and allele effects. This section describes the new development of this study that allows to calculate the variance among DH lines from any number of founding haplotypes. We start with describing the calculation from a quantitative genetic perspective using the infinitesimal model based on inbreeding and coancestry to give better intuition for the exact calculation based on QTL effects, haplotypes, and recombination frequencies, which is presented at the end of *Connection between infinitesimal model and haplotype-based approach*.

#### Quantitative genetic motivation

To illustrate the general principle, suppose 16 unrelated haplotypes A–P are available as founders from which DH offspring are derived. A crossing plan for these founders and their offspring, grandoffspring, and so forth could look like the plan depicted in Table 3. The crossing plan design is comparable to a multiparent advanced generation intercross population design, abbreviated ‘MAGIC’, as depicted in Fig. 1 of Scott *et al.* (2020) to which we refer the interested reader for visualization.

**Table 3.** Example crossing plan for haplotypes A–P.

Founder haplotypes																
	1	2	3	4	5	6	7	8	9	10	11	12	13	14	15	16
Gen0	AA	BB	CC	DD	EE	FF	GG	HH	II	JJ	KK	LL	MM	NN	OO	PP
Gen1	AxB		CxD		ExF		GxH		IxJ		KxL		MxN		OxP	
Gen2	ABxCD				EFxGH				IJxKL				MNxOP			
Gen3	ABCDxEFGH								IJKLxMNOP							
Gen4	ABCDEFGHxIJKLMNOP															
Gen5	DHs ABCDEFGHIJKLMNOP															

Groups of individuals shown as “ABxCD”, for example, refer to individuals produced by the gametes of “AxB” and “CxD”, i.e. recombination only occurs between haplotypes within a parent and no recombination occurs between chromosomes from the mother (“AxB”) and the father (“CxD”). The individuals “DHs ABCDEFGHIJKLMNOP” are DH lines produced from gametes of individuals of “ABCDEFGHxIJKLMNOP.”

The gametic MSV of each of the founders in Table 3 is 0 since they are all fully homozygous. Under the infinitesimal model, the gametic MSV of each of their descendants is assumed to be $0.25\sigma_{A}^{2}$, as they are all non-inbred given that the founders are all unrelated in this example. The only exception are the DH individuals in generation 5, whose gametic MSV is 0 because DHs are completely homozygous.

As a general principle, the genetic variance in each of the groups in the crossing plan up to generation 4 is determined by two factors. First, it includes the variance of the parent average BVs, which is calculated as the sum of the genetic variances of both parent groups divided by 4. Second, it also includes the gametic MSV of both parents, as expressed by Equation (2). The variances in the direct offspring groups of the founders, AxB, CxD, ExF, GxH, IxJ, KxL, MxN, and OxP, which plant breeders would call F1, or S0 (=0 rounds of selfing), is $0\sigma_{A}^{2}$. Each individual in generation 1 is expected to show a gametic variance of $0.25\sigma_{A}^{2}$. As a consequence, the within group variance in generation 2 is expected to be $0.5\sigma_{A}^{2}$. The variance of parent average BVs is $0.25\sigma_{A}^{2}$ for generation 3. The variance in each group in generation 3 is thus $0.75\sigma_{A}^{2}$. Following the same procedure, the genetic variance within each group in generation 4 is expected to be $0.875\sigma_{A}^{2}$. The genetic variance of DH lines produced by doubling the chromosomes of gametes produced by the group in generation 4 is $1.875\sigma_{A}^{2}$. This is because the individuals of generation 4 are not mated to each other. This means that the variance of parent average BVs is the same as the variance of group “ABCDEFGHxIJKLMNOP”, and the DH MSV is always 4 times larger than the gametic MSV (see Equation (4)). The variances for each group as well as their calculation are shown in Table 4. Following the presented principles, the calculation as shown in Table 4 can be extended to any number of generations. Table 4 also shows that the genetic variance of any group can be decomposed into gametic MSV of their ancestors.

**Table 4.** Genetic variances and gametic MSVs for each of the descendant's group types shown in Table 3.

Generation	Groups	Genetic variance	Gametic MSV
Gen0	AA, BB, CC, DD, EE, FF, GG, HH, II, JJ, KK, LL, MM, NN, OO, PP (founding haplotypes)	0	0
Gen1	AxB, CxD, ExF, GxH, IxJ, KxL, MxN, OxP (F1s)	$\frac{var(BV_{A})}{4}+\frac{var(BV_{B})}{4}+$ $\sigma_{gamMS_{AxA}}^{2}+\sigma_{gamMS_{BxB}}^{2}$ $\frac{0}{4}+\frac{0}{4}+0+0=0$	$\frac{1}{4}$
Gen2	ABxCD, EFxGH, IJxKL, MNxOP (F1s)	$\frac{var(BV_{AxB})}{4}+\frac{var(BV_{CxD})}{4}+$ $\sigma_{gamMS_{AxB}}^{2}+\sigma_{gamMS_{CxD}}^{2}$ $\frac{0}{4}+\frac{0}{4}+\frac{1}{4}+\frac{1}{4}=\frac{1}{2}=0.5$	$\frac{1}{4}$
Gen3	ABCDxEFGH, IJKLxMNOP (F1s)	$\frac{var(BV_{ABxCD})}{4}+\frac{var(BV_{EFxGH})}{4}+$ $\sigma_{gamMS_{ABxCD}}^{2}+\sigma_{gamMS_{EFxGH}}^{2}$ $\frac{0.5}{4}+\frac{0.5}{4}+\frac{1}{4}+\frac{1}{4}=\frac{6}{8}=0.75$	$\frac{1}{4}$
Gen4	ABCDEFGHxIJKLMNOP (F1s)	$\frac{var(BV_{ABCDxEFGH})}{4}+\frac{var(BV_{IJKLxMNOP})}{4}+$ $\sigma_{gamMS_{ABCDxEFGH}}^{2}+\sigma_{gamMS_{IJKLxMNOP}}^{2}$ $\frac{0.75}{4}+\frac{0.75}{4}+\frac{1}{4}+\frac{1}{4}=\frac{14}{16}=0.875$	$\frac{1}{4}$
Gen5	DHs ABCDEFGHIJKLMNOP (DHs)	$var(BV_{ABCDEFGHxIJKLMNOP})+$ $4\sigma_{gamMS_{ABCDEFGHxIJKLMNOP}}^{2}$ $0.875+4\frac{1}{4}=1.875$	0, not relevant

The genetic variance is the variance of BVs among members of the same group. The gametic MSV is the average gametic MSV of individuals of the group (e.g. the average of individuals of the 4-way cross ABxCD). Units are genetic variance $\sigma_{A}^{2}$. The individuals in generation 5 are DHs.

#### Connection between infinitesimal model and haplotype-based approach

As shown in Table 1, the matrix elements $\Sigma_{\;jl}\;$ of Σ can be calculated with $4(1-2c_{\;jl}^{\left(1\right)})D_{\;jl}^{AB}$ for the DH variance based on a 2-way cross. For brevity of the explanation here, we will write all crossover probabilities $c_{\;jl}^{\left(1\right)}$ in a matrix c and all linkage disequilibrium parameters between 2 parents $D_{\;jl}$ in a matrix D. Expressing the DH variance as $\sigma_{2-way\;DH}^{2}=\beta^{\prime }[4(11^{\prime }-2c)⊙D]\beta$ makes the equation easier to read. The term $11^{\prime }$ has dimensions pxp and is a matrix of ones, i.e. the outer product of a vector of ones. The “⊙” indicates the Hadamard product, i.e. element-wise matrix multiplication. To convert to gametic variance, division by 4 is required, so we can write $\sigma_{2-way\;F1\;gametes}^{2}=\beta^{\prime }[(11^{\prime }-2c)⊙D]\beta$. To give some interpretation, assume a trait is controlled by many loci that are all independent, i.e. unlinked. Then, the off-diagonal elements of *c*, so the recombination frequencies between loci, are 0.5 and the diagonal elements are 0. Consequently, the term $(11^{\prime }-2c)⊙D$ will result in a matrix of 0s, except for diagonal elements where values will be 0.25 in case haplotype A carries different alleles than haplotype B at the corresponding loci. The fraction of loci at which A and B are identical is the same as the homozygosity level of offspring produced by crossing A with B. In addition to the assumption that the trait is controlled by many loci, let us assume, for simplicity, that the beneficial alleles at all loci have the same effect size. Note that this is not necessarily the same assumption as in infinitesimal models (Walsh and Lynch 2018) but very similar. This assumption means that every heterozygous locus will create the same amount of variance in the gametes produced by an individual. Since we assumed independent loci in this example, there is no covariance between loci in the gametes. Therefore, the variances generated by all heterozygous loci can directly be summed to get the gametic MSV. Assume that all QTLs are used to measure the average heterozygosity, or assume that the subset of (marker) loci used to measure the homozygosity level (homozygosity = 1 − heterozygosity) represents the homozygosity level at QTL reasonably well. Then, it becomes clear that an individual's homozygosity level, or inbreeding level, is a perfect predictor for its gametic MSV. This is reflected in the expectation of the gametic MSV as $\sigma_{gamMS_{individual}}^{2}=0.25(1-F_{individual})\sigma_{A}^{2}$ (Dempfle 1990) under the infinitesimal model.

The inbreeding level of an individual resulting from the cross of A and B can be predicted from the coefficient of coancestry between A and B as $F_{AxB}=f_{AB}$ (Falconer and Mackay 1996, chapter 5). In the same way, the expected inbreeding coefficient of an individual ABxCD can be derived from the coancestry of its founders as $F_{ABxCD}=\frac{1}{4}(\;f_{AC}+f_{AD}+f_{BC}+f_{BD})$. That is, the inbreeding coefficient, and thus the gametic MSV, can be derived by consideration of the coancestries of the founders. What is shown here for ABxCD individuals can be extended to the inbreeding coefficient calculation of any future individual.

The prediction of genetic variance of 4-way DHs expressed based on coancestries of their founders is shown in Equation (8).


(8) $$\begin{array}{rl} & \sigma_{DH_{ABCD}}^{2}\\ & =4\sigma_{gamMS_{ABxCD}}^{2}+\sigma_{\;parent\;average_{ABxCD}}^{2}\\ & =\;4*0.25(1-{\bar{F}}_{ABxCD})\sigma_{A}^{2}+2*0.25\left(1-\frac{1}{2}(F_{AxB}+F_{CxD})\right)\sigma_{A}^{2}\\ & =4*0.25\left(1-\frac{1}{4}(\;f_{AC}+f_{AD}+f_{BC}+f_{BD})\right)\sigma_{A}^{2}\\ & \;+2*0.25\left(1-\frac{1}{2}(\;f_{AB}+f_{CD})\right)\sigma_{A}^{2} \end{array}$$

The genetic variance of DH families based on any number of founders can be predicted based on the coancestry between founders. Here, we show equations for 8-way crosses and 16-way crosses for the prediction based on inbreeding levels (Equations (9) and (10)). We also show the same equations based on coancestry between parents as the predictor of their offspring homozygosity level (Equations (11) and (12)). These equations help to visualize the pattern by which equations evolve for higher-order crosses and establish a better connection to haplotype-based predictions that will be presented later. The equations show that the number of coancestries required to derive the inbreeding level of F1s is growing exponentially (4 for 4-way F1, 16 for 8-way F1, and 64 for 16-way F1). Regardless of the number of coancestries, however, the expectation of the gametic MSV for a given inbreeding level is always the same (e.g. for F = 0, $0.25\sigma_{A}^{2}$). As shown with Equation (2) before, the variance among the parental average BVs of individuals in the offspring generation is, under random mating, half the genetic variance of the parental generation. This explains why the factor by which the gametic MSV is multiplied decays by a half with each step to an older generation starting from the *X*-way F1 generation (so from 2 to 1 to 0.5, and, although not shown, then 0.25, 0.125, and so on) (e.g. see Equation (12)). This factor ensures that the variance is estimated on the right level as can be noticed by considering Table 4. Table 4 shows that the expected variance increases asymptotically to 1 for F1s the more unrelated founders are involved in the cross (0, 0.5, 0.75, and 0.875 for F1s of 2-way, 4-way, 8-way, and 16-way crosses, respectively). By extension, the expected variance among DH offspring increases asymptotically to 2 (1, 1.5, 1.75, and 1.875 for DHs of 2-way, 4-way, 8-way, and 16-way crosses, respectively). Equations (8–12) match these expectations.


(9) $$\begin{array}{rl} & \sigma_{DH_{ABCDEFGH}}^{2}\\ & =4\sigma_{gamMS_{ABCDxEFGH}}^{2}+\sigma_{\;parent\;average_{ABCDxEFGH}}^{2}\\ & =\;4\;*\;0.25(1-{\bar{F}}_{ABCDxEFGH})\sigma_{A}^{2}\\ & \;+2\;*\;0.25\left(1-\frac{1}{2}({\bar{F}}_{ABxCD}+{\bar{F}}_{EFxGH})\right)\sigma_{A}^{2}\\ & \;+1\;*\;0.25\left(1-\frac{1}{2}(F_{AxB}+F_{CxD})\right)\sigma_{A}^{2} \end{array}$$

(10) $$\begin{array}{rl} & \sigma_{DH_{ABCDEFGHIJKLMNOP}}^{2}\\ & =\;4\;*\;0.25(1-{\bar{F}}_{ABCDEFGHxIJKLMNOP})\sigma_{A}^{2}\\ & \;+2\;*\;0.25\left(1-\frac{1}{2}({\bar{F}}_{ABCDxEFGH}+{\bar{F}}_{IJKLxMNOP})\right)\sigma_{A}^{2}\\ & \;+1\;*\;0.25\left(1-\frac{1}{4}({\bar{F}}_{ABxCD}+{\bar{F}}_{EFxGH}+{\bar{F}}_{IJxKL}+{\bar{F}}_{MNxOP})\right)\sigma_{A}^{2}\\ & \;+\frac{1}{2}\;*\;0.25\left(1-\frac{1}{8}(F_{AxB}+F_{CxD}+F_{ExF}+F_{GxH}+F_{IxJ}+F_{KxL}+F_{MxN}+F_{OxP})\right)\sigma_{A}^{2} \end{array}$$

(11) $$\begin{array}{rl} & \sigma_{DH_{ABCDEFGH}}^{2}\\ & =4\sigma_{gamMS_{ABCDxEFGH}}^{2}+\sigma_{\;parent\;average_{ABCDxEFGH}}^{2}\\ & =4\;*\;0.25\left(1-\frac{1}{16}\left(\begin{array}{c} \;f_{AE}+f_{AF}+f_{AG}+f_{AH}+f_{BE}+f_{BF}+f_{BG}+f_{BH}+\\ \;f_{CE}+f_{CF}+f_{CG}+f_{CH}+f_{DE}+f_{DF}+f_{DG}+f_{DH} \end{array}\right)\right)\sigma_{A}^{2}\\ & \;+2\;*\;0.25\left(1-\frac{1}{8}(\;f_{AC}+f_{AD}+f_{BC}+f_{BD}+f_{EG}+f_{EH}+f_{FG}+f_{FH})\right)\sigma_{A}^{2}\\ & \;+1\;*\;0.25\left(1-\frac{1}{4}(\;f_{AB}+f_{CD}+f_{EF}+f_{GH})\right)\sigma_{A}^{2} \end{array}$$

(12) $$\begin{array}{rl} & \sigma_{DH_{ABCDEFGHIJKLMNOP}}^{2}\\ & =4\sigma_{gamMS_{ABCDEFGHxIJKLMNOP}}^{2}+\sigma_{\;parent\;average_{ABCDEFGHxIJKLMNOP}}^{2}\\ & =\;4\;*\;0.25\left(1-\frac{1}{64}\left(\begin{array}{c} \;f_{AI}+f_{AJ}+f_{AK}+f_{AL}+f_{AM}+f_{AN}+f_{AO}+f_{AP}+\\ f_{BI}+f_{BJ}+f_{BK}+f_{BL}+f_{BM}+f_{BN}+f_{BO}+f_{BP}+\\ f_{CI}+f_{CJ}+f_{CK}+f_{CL}+f_{CM}+f_{CN}+f_{CO}+f_{CP}+\\ f_{DI}+f_{DJ}+f_{DK}+f_{DL}+f_{DM}+f_{DN}+f_{DO}+f_{DP}+\\ f_{EI}+f_{EJ}+f_{EK}+f_{EL}+f_{EM}+f_{EN}+f_{EO}+f_{EP}+\\ f_{FI}+f_{FJ}+f_{FK}+f_{FL}+f_{FM}+f_{FN}+f_{FO}+f_{FP}+\\ f_{GI}+f_{GJ}+f_{GK}+f_{GL}+f_{GM}+f_{GN}+f_{GO}+f_{GP}+\\ f_{HI}+f_{HJ}+f_{HK}+f_{HL}+f_{HM}+f_{HN}+f_{HO}+f_{HP} \end{array}\right)\right)\sigma_{A}^{2}\\ & \;+2\;*\;0.25\left(1-\frac{1}{32}\left(\begin{array}{c} \;f_{AE}+f_{AF}+f_{AG}+f_{AH}+f_{BE}+f_{BF}+f_{BG}+f_{BH}+\\ f_{CE}+f_{CF}+f_{CG}+f_{CH}+f_{DE}+f_{DF}+f_{DG}+f_{DH}+\\ f_{IM}+f_{IN}+f_{IO}+f_{IP}+f_{JM}+f_{JN}+f_{JO}+f_{JP}+\\ f_{KM}+f_{KN}+f_{KO}+f_{KP}+f_{LM}+f_{LN}+f_{LO}+f_{LP} \end{array}\right)\right)\sigma_{A}^{2}\\ & \;+1\;*\;0.25\left(1-\frac{1}{16}\left(\begin{array}{c} \;f_{AC}+f_{AD}+f_{BC}+f_{BD}+f_{EG}+f_{EH}+f_{FG}+f_{FH}+\\ \;f_{IK}+f_{IL}+f_{JK}+f_{JL}+f_{MO}+f_{MP}+f_{NO}+f_{NP} \end{array}\right)\right)\sigma_{A}^{2}\\ & \;+\frac{1}{2}\;*\;0.25\left(1-\frac{1}{8}(\;f_{AB}+f_{CD}+f_{EF}+f_{GH}+f_{IJ}+f_{KL}+f_{MN}+f_{OP})\right)\sigma_{A}^{2} \end{array}$$

Until here, we assumed that all loci are independent so that we could pretend that $(1-4av(diag(D^{AB})))=f_{AB}$, i.e. the coancestry is perfectly correlated to the average entry on the diagonal in the disequilibrium matrix. In reality, alleles are linked, which is ignored by inbreeding or coancestry-based predictions. Their poor predictive ability was reported in dairy cattle (Segelke *et al.* 2014; Santos *et al.* 2019). Thus, linkage disequilibrium, which causes covariance between alleles in descendants, should not be ignored. To account for linkage, one needs to make the following modification to the coancestry-based equations above: swap the coancestry terms ($(1-0.25f)\sigma_{A}^{2}$) with the respective disequilibrium indicators (D) multiplied with the allele effect of the paternal haplotype ($\beta_{pat}$) and the maternal haplotype ($\beta_{mat}$), which for simplicity we assume to be identical here $\left(\beta_{pat}=\beta_{mat}\right)$, multiplied with $(11^{\prime }-2c{)}^{m}$, where *m* is the number of meiosis, to account for linkage disequilibrium decay by recombination. In short, swap $(1-0.25f)\sigma_{A}^{2}$ with $\beta^{\prime }[{\left(11^{\prime }-2c\right)}^{m}⊙D]\beta$.

The result of the modifications for an 8-way cross is shown in Equation (13).


(13) $$\begin{array}{rl} & \sigma_{DH_{ABCDEFGH}}^{2}\\ & =4*\left(\frac{1}{16}\left(\begin{array}{c} \beta^{\prime }[{\left(11^{\prime }-2c\right)}^{1}⊙D^{AE}]\beta +\beta^{\prime }[{\left(11^{\prime }-2c\right)}^{1}⊙D^{AF}]\beta +\beta^{\prime }[{\left(11^{\prime }-2c\right)}^{1}⊙D^{AG}]\beta +\beta^{\prime }[{\left(11^{\prime }-2c\right)}^{1}⊙D^{AH}]\beta +\\ \beta^{\prime }[{\left(11^{\prime }-2c\right)}^{1}⊙D^{BE}]\beta +\beta^{\prime }[{\left(11^{\prime }-2c\right)}^{1}⊙D^{BF}]\beta +\beta^{\prime }[{\left(11^{\prime }-2c\right)}^{1}⊙D^{BG}]\beta +\beta^{\prime }[{\left(11^{\prime }-2c\right)}^{1}⊙D^{BH}]\beta +\\ \beta^{\prime }[{\left(11^{\prime }-2c\right)}^{1}⊙D^{CE}]\beta +\beta^{\prime }[{\left(11^{\prime }-2c\right)}^{1}⊙D^{CF}]\beta +\beta^{\prime }[{\left(11^{\prime }-2c\right)}^{1}⊙D^{CG}]\beta +\beta^{\prime }[{\left(11^{\prime }-2c\right)}^{1}⊙D^{CH}]\beta +\\ \beta^{\prime }[{\left(11^{\prime }-2c\right)}^{1}⊙D^{DE}]\beta +\beta^{\prime }[{\left(11^{\prime }-2c\right)}^{1}⊙D^{DF}]\beta +\beta^{\prime }[{\left(11^{\prime }-2c\right)}^{1}⊙D^{DG}]\beta +\beta^{\prime }[{\left(11^{\prime }-2c\right)}^{1}⊙D^{DH}]\beta \end{array}\right)\right)\\ & \;+2*\left(\frac{1}{8}\left(\begin{array}{c} \beta^{\prime }[{\left(11^{\prime }-2c\right)}^{2}⊙D^{AC}]\beta +\beta^{\prime }[{\left(11^{\prime }-2c\right)}^{2}⊙D^{AD}]\beta +\beta^{\prime }[{\left(11^{\prime }-2c\right)}^{2}⊙D^{BC}]\beta +\beta^{\prime }[{\left(11^{\prime }-2c\right)}^{2}⊙D^{BD}]\beta +\\ \beta^{\prime }[{\left(11^{\prime }-2c\right)}^{2}⊙D^{EG}]\beta +\beta^{\prime }[{\left(11^{\prime }-2c\right)}^{2}⊙D^{EH}]\beta +\beta^{\prime }[{\left(11^{\prime }-2c\right)}^{2}⊙D^{FG}]\beta +\beta^{\prime }[{\left(11^{\prime }-2c\right)}^{2}⊙D^{FH}]\beta \end{array}\right)\right)\\ & \;+1*\left(\frac{1}{4}(\beta^{\prime }[{\left(11^{\prime }-2c\right)}^{3}⊙D^{AB}]\beta +\beta^{\prime }[{\left(11^{\prime }-2c\right)}^{3}⊙D^{CD}]\beta +\beta^{\prime }[{\left(11^{\prime }-2c\right)}^{3}⊙D^{EF}]\beta +\beta^{\prime }[{\left(11^{\prime }-2c\right)}^{3}⊙D^{GH}]\beta )\right) \end{array}$$

Equation (13) can be simplified further to improve readability and to reduce the number of matrix multiplications which consequently reduces computation time. First, the allele effect vectors β can be removed so that $(11^{\prime }-2c{)}^{m}⊙D$ calculations can be made and summed up. The sum, which is then the matrix Σ, can be multiplied with the QTL effect vector β so that $\sigma_{DH\;Progeny}^{2}=\beta^{\prime }\;\Sigma \beta$. Second, all D matrices that are to be multiplied with the same linkage disequilibrium decay matrix , i.e. matrices with the same power of *m*, can be summed up. Lastly, the factors like $\frac{1}{16}$, $\frac{1}{8}$, and $\frac{1}{4}$, which express nothing other than that the variances are to be averaged, can be combined with the factors (4, 2, and 1) in front of the parentheses that scale the average gametic MSVs to the right level. For the 8-way cross, their multiplications are $\frac{4}{16}$, $\frac{2}{8}$, and $\frac{1}{4}$, i.e. the results are all identical. These factor multiplications are also all identical for 4-way, 16-way, or any other *X*-way cross. They only depend on the number of founding haplotypes. The general expression based on the number of haplotypes is thus ${\left(\frac{4}{\mathrm{number}\;\mathrm{of}\;\mathrm{founder}\;\mathrm{haplotypes}}\right)}^{2}$. Altogether, for the prediction of the matrix element $\Sigma_{\;jl}$ for an 8-way cross, we arrive at Equation (14), and at Equation (15) for a 16-way cross.


(14) $$\Sigma_{\;jl}=\left(\begin{array}{c} (1-2c_{\;jl}^{\left(1\right)}{)}^{1}\left(\begin{array}{c} D_{\;jl}^{AE}+D_{\;jl}^{AF}+D_{\;jl}^{AG}+D_{\;jl}^{AH}+D_{\;jl}^{BE}+D_{\;jl}^{BF}+D_{\;jl}^{BG}+D_{\;jl}^{BH}+\\ D_{\;jl}^{CE}+D_{\;jl}^{CF}+D_{\;jl}^{CG}+D_{\;jl}^{CH}+D_{\;jl}^{DE}+D_{\;jl}^{DF}+D_{\;jl}^{DG}+D_{\;jl}^{DH} \end{array}\right)+\\ (1-2c_{\;jl}^{\left(1\right)}{)}^{2}(D_{\;jl}^{AC}+D_{\;jl}^{AD}+D_{\;jl}^{BC}+D_{\;jl}^{BD}+D_{\;jl}^{EG}+D_{\;jl}^{EH}+D_{\;jl}^{FG}+D_{\;jl}^{FH})+\\ \left(1-2c_{\;jl}^{\left(1\right)}{)}^{3}(D_{\;jl}^{AB}+D_{\;jl}^{CD}+D_{\;jl}^{EF}+D_{\;jl}^{GH}\right) \end{array}\right)*{\left(\frac{4}{8}\right)}^{2}\;(14)$$

(15) $$\Sigma_{\;jl}=\left(\begin{array}{c} (1-2c_{\;jl}^{\left(1\right)}{)}^{1}\left(\begin{array}{c} D_{\;jl}^{AI}+D_{\;jl}^{AJ}+D_{\;jl}^{AK}+D_{\;jl}^{AL}+D_{\;jl}^{AM}+D_{\;jl}^{AN}+D_{\;jl}^{AO}+D_{\;jl}^{AP}+\\ D_{\;jl}^{BI}+D_{\;jl}^{BJ}+D_{\;jl}^{BK}+D_{\;jl}^{BL}+D_{\;jl}^{BM}+D_{\;jl}^{BN}+D_{\;jl}^{BO}+D_{\;jl}^{BP}+\\ D_{\;jl}^{CI}+D_{\;jl}^{CJ}+D_{\;jl}^{CK}+D_{\;jl}^{CL}+D_{\;jl}^{CM}+D_{\;jl}^{CN}+D_{\;jl}^{CO}+D_{\;jl}^{CP}+\\ D_{\;jl}^{DI}+D_{\;jl}^{DJ}+D_{\;jl}^{DK}+D_{\;jl}^{DL}+D_{\;jl}^{DM}+D_{\;jl}^{DN}+D_{\;jl}^{DO}+D_{\;jl}^{DP}+\\ D_{\;jl}^{EI}+D_{\;jl}^{EJ}+D_{\;jl}^{EK}+D_{\;jl}^{EL}+D_{\;jl}^{EM}+D_{\;jl}^{EN}+D_{\;jl}^{EO}+D_{\;jl}^{EP}+\\ D_{\;jl}^{FI}+D_{\;jl}^{FJ}+D_{\;jl}^{FK}+D_{\;jl}^{FL}+D_{\;jl}^{FM}+D_{\;jl}^{FN}+D_{\;jl}^{FO}+D_{\;jl}^{FP}+\\ D_{\;jl}^{GI}+D_{\;jl}^{GJ}+D_{\;jl}^{GK}+D_{\;jl}^{GL}+D_{\;jl}^{GF}+D_{\;jl}^{GN}+D_{\;jl}^{GO}+D_{\;jl}^{GP}+\\ D_{\;jl}^{HI}+D_{\;jl}^{HJ}+D_{\;jl}^{HK}+D_{\;jl}^{HL}+D_{\;jl}^{HM}+D_{\;jl}^{HN}+D_{\;jl}^{HO}+D_{\;jl}^{HP} \end{array}\right)+\\ (1-2c_{\;jl}^{\left(1\right)}{)}^{2}\left(\begin{array}{c} D_{\;jl}^{AE}+D_{\;jl}^{AF}+D_{\;jl}^{AG}+D_{\;jl}^{AH}+D_{\;jl}^{BE}+D_{\;jl}^{BF}+D_{\;jl}^{BG}+D_{\;jl}^{BH}+\\ D_{\;jl}^{CE}+D_{\;jl}^{CF}+D_{\;jl}^{CG}+D_{\;jl}^{CH}+D_{\;jl}^{DE}+D_{\;jl}^{DF}+D_{\;jl}^{DG}+D_{\;jl}^{DH}+\\ D_{\;jl}^{IM}+D_{\;jl}^{IN}+D_{\;jl}^{IO}+D_{\;jl}^{IP}+D_{\;jl}^{JM}+D_{\;jl}^{JN}+D_{\;jl}^{JO}+D_{\;jl}^{JP}+\\ D_{\;jl}^{KM}+D_{\;jl}^{KN}+D_{\;jl}^{KO}+D_{\;jl}^{KP}+D_{\;jl}^{LM}+D_{\;jl}^{LN}+D_{\;jl}^{LO}+D_{\;jl}^{LP} \end{array}\right)+\\ (1-2c_{\;jl}^{\left(1\right)}{)}^{3}\left(\begin{array}{c} D_{\;jl}^{AC}+D_{\;jl}^{AD}+D_{\;jl}^{BC}+D_{\;jl}^{BD}+D_{\;jl}^{EG}+D_{\;jl}^{EH}+D_{\;jl}^{FG}+D_{\;jl}^{FH}+\\ D_{\;jl}^{IK}+D_{\;jl}^{IL}+D_{\;jl}^{JK}+D_{\;jl}^{JL}+D_{\;jl}^{MO}+D_{\;jl}^{MP}+D_{\;jl}^{NO}+D_{\;jl}^{NP} \end{array}\right)+\\ \left(1-2c_{\;jl}^{\left(1\right)}{)}^{4}(D_{\;jl}^{AB}+D_{\;jl}^{CD}+D_{\;jl}^{EF}+D_{\;jl}^{GH}+D_{\;jl}^{IJ}+D_{\;jl}^{KL}+D_{\;jl}^{MN}+D_{\;jl}^{OP}\right) \end{array}\right)*{\left(\frac{4}{16}\right)}^{2}\;(15)$$

In general, the approach to calculate the variance among DH lines derived from any number of founding haplotypes can be calculated as presented in Equation (16) below. In words, the number of founders allowed to be used in the equation is 1, 2, 4, 8, 16, 32, 64, and so on. $m_{ik}$ indicates how many meiotic events are in between the generation of DH lines and the generation in which the allele at locus *j* derived from founder *i* and the allele at locus *l* derived from founder *k* could have been combined in the same individual. Note that the number of founders required as input for Equation 16 is out of the set of $2^{m}$ but that does not mean that all founders actually have to be unique. For example, if the phased genotype of the same homozygous line is used at founder positions BB, CC, and DD (compare Table 3), then this is like backcrossing the offspring AxB back to BB (BB is identical to genotypes used at positions for CC and DD here). Also note that any degree of kinship between the founders is possible for use of the equation.


(16) $$\begin{array}{rl} & \Sigma_{\;jl}={\left(\frac{4}{N}\right)}^{2}\overset{N}{\underset{i=1}{\sum }}\;\overset{N}{\underset{k>i}{\sum }}\;(1-2c_{\;jl}^{\left(1\right)}{)}^{m_{ik}}D_{\;jl}^{ik} \end{array}$$

with {(*i*, *k*)|*i*, *k* ∈ {1, 2, 3, 4, . . .*N*}, *k* > *i*}, the number of founder haplotypes *N* as an element of 2^*m*^*m* ∈ *Z*^+^, and with *m* meiotic events between the generation in which haplotypes *i* and *k* could be combined in a genome and the generation of DHs.

### Conversion to gametic MSV

Most users are likely not interested in the variance of DH lines expected from a high order cross but rather in the expected gametic MSV of an individual in a future generation. To convert the variances expected among DH lines into the expected gametic MSV of a single individual, the same calculations as in Table 4 need to be done. For example, suppose the same crossing plan as in Table 3 shall be done and suppose the interest is in the average expected gametic MSV of individuals ABCDEFGHxIJKLMNOP. Then, the variances of DH lines produced by every group of individuals shown in Table 3 need to be calculated with the corresponding analytical equation. Based on the DH variances, the gametic MSVs can be worked out as shown in Table 5 for the case of 16 founding haplotypes. A numerical example is given in Supplementary Material 1.

**Table 5.** Calculation of gametic MSV for a random individual of a group based on calculated DH variances.

	Calculated DH variance	Gametic MSV
Generation 0	0	0
Generation 1	$\sigma_{2-way\;DH\;AB}^{2}$	$\sigma_{gamMS_{AxB}}^{2}$ $=\frac{\sigma_{2-way\;DH\;AB}^{2}}{4}$
Generation 2	$\sigma_{4-way\;DH\;ABCD}^{2}$	$\sigma_{gamMS_{ABxCD}}^{2}$ $=\frac{\left(\sigma_{4-way\;DH\;ABCD}^{2}-\sigma_{4-way\;F1\;ABxCD}^{2}\right)}{4}$ $\sigma_{4-way\;F1\;ABxCD}^{2}=\sigma_{gamMS_{AxB}}^{2}+\sigma_{gamMS_{CxD}}^{2}$
Generation 3	$\sigma_{8-way\;DH\;ABCDEFGH}^{2}$	$\sigma_{gamMS_{ABCDxEFGH}}^{2}$ $=\frac{\left(\sigma_{8-way\;DH\;ABCDEFGH}^{2}-\sigma_{8-way\;F1\;ABCDxEFGH}^{2}\right)}{4}$ $\sigma_{8-way\;F1\;ABCDxEFGH}^{2}$ $=\frac{\sigma_{4-way\;F1\;ABxCD}^{2}}{4}+\frac{\sigma_{4-way\;F1\;EFxGH}^{2}}{4}$ $+\;\sigma_{gamMS_{ABxCD}}^{2}+\sigma_{gamMS_{EFxGH}}^{2}$
Generation 4	$\sigma_{16-way\;DH\;ABCDEFGHIJKLMNOP}^{2}$	$\sigma_{gamMS_{ABCDEFGHxIJKLMNOP}}^{2}$ $=\frac{\left(\sigma_{16-way\;DH\;ABCDEFGHIJKLMNOP}^{2}-\sigma_{16-way\;F1\;ABCDEFGHxIJKLMNOP}^{2}\right)}{4}$ $\sigma_{16-way\;F1\;ABCDEFGHxIJKLMNOP}^{2}$ $=\frac{\sigma_{8-way\;F1\;ABCDxEFGH}^{2}}{4}+\frac{\sigma_{8-way\;F1\;IJKLxMNOP}^{2}}{4}$ $+\;\sigma_{gamMS_{ABCDxEFGH}}^{2}+\sigma_{gamMS_{IJKLxMNOP}}^{2}$

Only one group per generation is shown as an example.

### Validation with simulation

#### Scenario without selection

To confirm that the equations accurately describe the intended property, we conducted a simulated experiment. We compared the outcomes based on simulated progeny with the prediction based on our developed equation for (1) the genetic variance among DH lines of a 16-way cross, (2) the gametic MSV of 16-way F1 individuals, (3) the gametic Mendelian sampling covariance between two correlated traits in the gametes produced by 16-way F1 individuals, and (4) the correlation between two traits in the gametes produced by 16-way F1 individuals.

We simulated 100 16-way crosses using the same genetic linkage map and 57 real Iodent maize inbred lines as founder haplotypes as used by Allier, Moreau, *et al.* (2019) and provided in their Supplementary File S4. More information about the data set is not necessary for the purpose of the simulation in this study but the interested reader is referred to the paper of Allier, Moreau, *et al.* (2019) for a detailed description. This genome model is referred to as “corn” in our study.

We used the simulation software MoBPS version 1.11.40 of Pook *et al.* (2020). By default, MoBPS uses Haldane's mapping function to convert genetic distances to recombination rates. We modeled 2 arbitrary traits each controlled by 500 additive QTLs. QTL effects were drawn so that the correlation between the traits roughly matched 0.7. The genetic variance of trait 1 was arbitrarily chosen as 1, and the genetic variance of trait 2 was set to 100. For each of the 100 16-way crosses, 10,000 individuals were simulated for every group shown in Table 3. Mating between members of different groups was random. In addition, every individual only had 1 offspring to reduce drift due to variation in parental contributions and thus to reduce deviations from the expected outcome without drift. For example, 10,000 individuals were simulated for groups AxB and CxD each. To form group (AxB)x(CxD), an individual of group AxB was mated with a random individual of group CxD to produce 1 offspring and these individuals were never used as parents again. The process was repeated for all remaining 9,999 individuals of group (AxB)x(CxD).

Genetic covariances were calculated analytically by multiplying matrix element $\Sigma_{\;jl}$ with the respective allele effects of both traits instead of 2 times the effect of the same trait as one would do for genetic variances ($COV_{Progeny}(Trait1,\;Trait2)\;=\beta_{Trait1}^{\prime }\;\Sigma \beta_{Trait2}$, with $\beta_{Trait1}$ and $\beta_{Trait2}$ as vectors with allele substitution effects of trait 1 and 2). When using the analytical approach, the correlations were calculated based on the derived variances and covariances of the traits: $COR(Trait1,\;Trait2)=\;\frac{COV(Trait1,\;Trait2)}{\sqrt{VAR(Trait1)VAR(Trait2)}}$.

#### Scenario with selection

The setup above aims to validate our algebraic approach with simulated data. From a practical perspective, the most interesting application of gametic MSV would likely be the inclusion in a selection criterion. The gametic MSVs calculated based on equations developed in this study are an expectation for a random individual that has been produced from unselected ancestors by random mating. Here, “unselected” refers to focal individuals and all ancestors between the focal individuals and the founders, but not to the founders themselves as these may be selected. However, breeding programs do select every generation. Since selection can change the linkage disequilibrium and allele frequencies, gametic MSVs of individuals produced by selected parents may deviate from the prediction under the assumption of no selection, i.e. the assumption that allele frequencies do not change and that only recombination influences linkage disequilibrium.

To check the predictive ability of gametic MSV in case selection is applied in every generation, we conducted a second experiment similar to the first one with the exception that 60,000 individuals were simulated per group and the best 3,000 of every group were selected (5% selected) for the first trait. The predictive ability may be lower compared to the 10,000-individual scenario simply because less individuals are selected. To account for potentially lower predictive ability due to a lower number of individuals, we simulated the same crossing scheme again without selection and only 3,000 individuals in every group. We only report the gametic MSV of this second experiment.

We hypothesized that the predictive ability will be higher the more QTLs are segregating independently because the allele frequencies would change less since a genome with more effectively independent QTL is closer to the assumption made under the infinitesimal model. All QTLs on the same chromosome are linked to some extent, which implies that the number of QTL in a genome that is effectively independent is less than the actual number of QTL. If one imagined all QTL on a chromosome to be perfectly linked in a crude approximation, one can see each chromosome as a QTL. Thus, the corn genome would effectively only have 10 independent loci because of its 10 chromosomes. To investigate the influence of the deviation from the infinitesimal model, we repeated the same analysis with a genome that has 3 times the number of chromosomes as corn. We call this the “cattle” scenario hereafter as cattle have 30 chromosome pairs if including sex chromosomes. This artificial cattle genome was created by taking the genome of corn and tripling it, i.e. all SNPs on chromosome 1 have the same genetic map position, allele frequency, and linkage disequilibrium as those on chromosomes 11 and 21. For the first 10 chromosomes of cattle individuals, chromosomes 1–10 of the 57 corn individuals were used, i.e. the cattle and corn individuals are identical in their first 10 chromosomes. For chromosomes 11–30, the respective relevant chromosome (linkage group) was sampled from the 57 corn individuals randomly without replacement. We decided to use this reshuffling strategy to avoid creating additional artificial between-chromosome linkage disequilibrium of loci that are actually just copies of a single locus in the corn genome model. The allele substitution effects were multiplied by $\sqrt{1/3}$ so that the gametic MSVs obtained with the cattle genome model roughly match the level of the corn genome. This was done for display purposes.

To understand the differences between the corn and cattle scenario, we did some simple analysis of (1) the change of frequency of the beneficial allele, (2) the average homozygosity level, and (3) the change in within-chromosome linkage disequilibrium. For this analysis, only QTLs that were segregating among the 16 founders of each cross were included and results averaged over all 100 16-way crosses.

The change in allele frequency is reported as the average difference between the frequency of the beneficial allele in the last generation (RANDOM selection scenario), or the selected individuals in the last generation (SELECTED scenario), to the frequency in the founders ($AF_{\;focal\;individuals}-AF_{\;founder}$). The reported homozygosity level is the average percentage of QTLs that are homozygous.

The linkage disequilibrium between two loci was calculated according to Falconer and Mackay (1996) as $D=f_{AB}f_{ab}-f_{Ab}f_{aB}$, with $f_{AB}$ ($f_{ab}$) as the frequency of haplotypes with two beneficial (deleterious) alleles, and $f_{Ab}$ and $f_{aB}$ as the frequencies of haplotypes with the beneficial and deleterious alleles in repulsion phase. *D* has a positive (negative) sign when more (less) haplotypes are in coupling phase than expected under linkage equilibrium. Positive disequilibrium causes a higher genetic variance than expected under linkage equilibrium (Walsh and Lynch 2018). Our interest for analyzing the LD was to see if selection has a noticeable effect on LD which in turn would influence the gametic MSV of selected individuals. LD between loci located on different chromosomes does not impact the gametic MSV of an individual as the alleles will be unlinked in the gametes. Only within-chromosome LD cannot be entirely broken down by meiosis. Thus, we only analyzed the within-chromosome LD. We report the difference in average LD between individuals of the last generation of the RANDOM selection scenario and unselected individuals in the last generation of the SELECTED scenario (${\bar{D}}_{last\;generation\;SELECTED}-{\bar{D}}_{last\;generation\;RANDOM}$).

## Results

### Scenario without selection

**Fig. 2.**
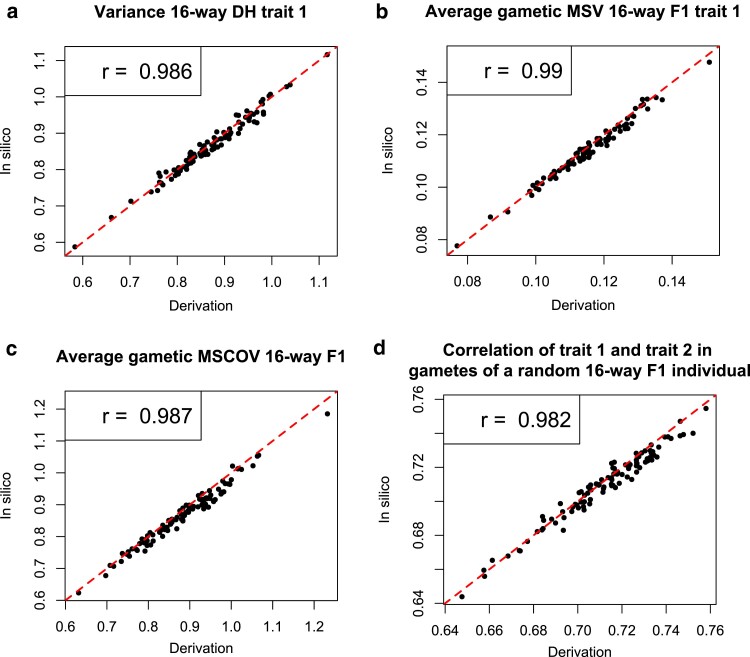
Comparison between analytically predicted (derivation) and empirical (in silico) properties of descendant individuals of 100 16-way crosses in which 10,000 individuals were simulated at every crossing step. Shown are a) the variances of BVs of trait 1 for each of the 16-way DH descendant groups; b) the average gametic MSV of trait 1 of 16-way F1 descendants; c) the average gametic Mendelian sampling covariance of trait 1 and trait 2 of 16-way F1 descendants; and d) the correlation of BVs for trait 1 and trait 2 in gametes produced by a 16-way random F1 descendant. Correlations between predicted and empirical properties are indicated within each plot. The red dashed lines have an intercept of 0 and a slope of 1 to aid visualization.

The predictions based on analytical equations matched the simulation-based predictions very well with correlations of all investigated variables above 0.98 (Fig. 2). Note that not only all correlations are high but also the range of values of the in silico predictions and analytical predictions match very well. In some plots of Fig. 2, more than 50% of the points appear to be below the indicated ideal regression lines, meaning that the algebraic method predicts slightly higher values than expected from the simulation-based approach. This discrepancy is small with the simulation-based predictions being on average 0.46%, 0.95%, 1.83%, and 0.35% ($\frac{av(V_{{DH}_{analytical}})\;-\;av(V_{{DH}_{simulated}})}{av(V_{{DH}_{analytical}})}*100\text{\%}$ ) lower than the analytical predictions for the DH variance, the 16-way F1 gametic MSV, the gametic MS covariance, and the gametic MS correlation (plot A to plot D in Fig. 2), respectively. We hypothesize that this small discrepancy is the result of noise of the stochastic process of simulation. Thus, we conclude that the analytical approach not only has a high predictive power but accurately describes the property it was designed to measure.

### Scenario with selection

**Fig. 3.**
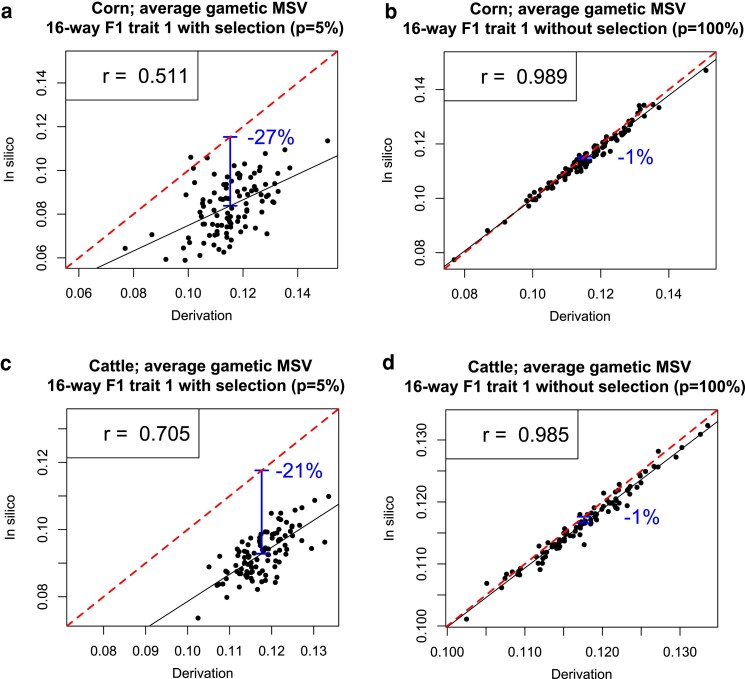
Comparison between analytically predicted (derivation) gametic MSV, which does not consider selection, and observed gametic MSV (in silico) of 16-way F1 individuals for 100 16-way crosses when selection is applied in every group a, c) and without selection b, d). Solid black lines indicate estimated regression lines. Blue arrows highlight the difference between the average analytical and empirical prediction. The difference in percentage is indicated in blue. Correlations between predicted and empirical properties are indicated within each plot. The red dashed lines have an intercept of 0 and a slope of 1 to aid visualization.

The predictive correlation for the gametic MSV of selected 16-way F1 individuals produced from selected 8-way F1 individuals, which are themselves produced from selected 4-way F1 individuals, had a value of 0.511 and 0.705 for the corn and cattle genome, respectively. This was considerably lower than in the case in which no selection was applied (0.989 and 0.985) (see Fig. 3). Furthermore, the discrepancy between the analytical prediction and the observed gametic MSV of individuals produced from selected ancestors was substantially higher: 27% for corn and 21% for cattle, compared to just 1% in the scenario without selection.

The distribution of points in the cloud of the 3,000-individual setup for corn (plot B in Fig. 3) is very similar to the distribution in the 10,000-individual setup (plot B in Fig. 2) because the simulated crossing scheme was the same. The slightly lower predictive correlation in the 3,000-individual scenario compared to the 10,000-individual setup may be attributed to a slightly higher sampling error due to the smaller group sizes but is not appreciable.

The predictive correlation for the gametic MSV of cattle was much better than the one for corn in case individuals were selected (compare plot A with plot C in Fig. 3). The change in allele frequency and the homozygosity levels were not significantly different between the corn and cattle scenario when no selection was applied (Welch 2-sample *t*-test, function “t.test()” in R). However, in the SELECTED scenario, the allele frequencies changed much more for both genomes leading to higher homozygosity levels, as shown in Table 6. Relative to the RANDOM scenario, the individuals of the SELECTED scenario also showed more negative linkage disequilibrium between beneficial loci. Overall, the changes induced by selection seem to affect the corn genome stronger than the cattle genome with *P*-values of the *t*-test being much lower than 0.001.

**Table 6.** Average allele frequency change and homozygosity level for the tested scenarios as well as the difference in mean disequilibrium between beneficial alleles between RANDOM and selection scenarios.

	Allele frequency change (in percentage points)		Homozygosity level (%)		Difference in disequilibrium
	RANDOM	SELECTED	RANDOM	SELECTED	
Corn	0.006	5.2	68.1	71.0	−0.00027
Cattle	−0.003	3.1	67.8	69.7	−0.00014
Difference between corn and cattle	n.s.	*	n.s.	*	*

Significance levels are indicated with “n.s.” for non-significant (*P* > 0.05) and “*” for significant with *P*-values lower than 0.001.

We were interested to what extend the lower prediction accuracy for the gametic MSV of selected 16-way F1 individuals would lead to different selection decisions. For this comparison, we used data from the scenario with selection. The gametic MSV of 16-way F1 individuals and the gametic MSV of all their ancestors were predicted with the presented analytical approach (“Derivation” in Table 7). These variances were used as input to calculate the *ExpBVSelGrGrGrOff* criterion, and the result was compared to the average genetic level of selected individuals in the F4 that were produced by mating selected “F1 ABCDEFGHxIJKLMNOP” at random in the simulation. The Pearson correlation coefficient and Spearman's rank correlation coefficient between the observed genetic level and the level predicted by the *ExpBVSelGrGrGrOff* criterion as well as the parent average BV are shown in Table 7.

**Table 7.** Pearson’s correlation coefficient and Spearman's rank correlation coefficient between different predictors and the observed genetic level in 16-way F1 individuals when the 5% best are selected every generation.

		Observed genetic level	
		Pearson correlation	Spearman correlation
Corn	Derivation	0.899	0.865
	Parent average BV	0.693	0.634
Cattle	Derivation	0.974	0.966
	Parent average BV	0.858	0.826

Despite the intermediate accuracy (0.511 for corn, 0.705 for cattle) for the prediction of gametic MSV of selected 16-way F1 individuals with the presented analytical approach, the prediction accuracy for the genetic level (Pearson correlation) and the ranking (Spearman correlation) was still high (>0.86). This means that some sets of 16-founder combinations will be selected that would not be selected if selection was accounted for in the variance prediction. However, the difference in selection decisions in likely to be relatively small. Using gametic MSV predicted under the assumption of no selection for the *ExpBVSelGrGrGrOff* criterion resulted in a small overestimation of genetic gain (Supplementary Material 3), which is due to gametic MSV of selected individuals being smaller than that of unselected ones (Fig. 3). The parent average BV appears to be an intermediate (corn) to good (cattle) predictor of the genetic level in the F4 generation. For breeding programs, this means that e.g. preselection decisions can be made based on estimated BVs while only using the *ExpBVSelGrGrGrOff* criterion for final decisions. This would reduce the complexity by only evaluating a limited set of founders, while the preselection step is not expected to cause the final selection decisions to be very different. Visualization of this small experiment can be found in Supplementary Material 3.

## Discussion

Approaches for predicting genetic variance of descendants (Bonk *et al.* 2016; Lehermeier *et al.* 2017; Osthushenrich *et al.* 2017; Allier, Moreau, *et al.* 2019; Wolfe *et al.* 2021) or gametes (Santos *et al.* 2019; Musa and Reinsch 2023) based on expected recombination frequencies and haplotype information of parents have been published in recent years. All previous approaches are limited in that they can only consider 2 or at most 4 haplotypes. This limitation has been lifted with the approach presented here that allows to consider any number of founder haplotypes. Note that although the number of haplotypes required as input in Equation (16) is a power of 2, in principle, any number of haplotypes can be evaluated. If less lines shall be used than required, for inbred lines, the actual haplotypes may be used in positions for founders “AA” and “BB” as the outcome AxB is identical to the parent. For non-fully inbred individuals, the paternal haplotype may be used in positions for founders “AA” and “BB” and the maternal one in positions for “CC” and “DD” for example.

We compared results of the analytical derivation with simulated individuals and found very high correlations (all above 0.98, see Fig. 2). The correlations are not 1 because of Monte Carlo errors, or in genetic terms, because of genetic drift in the simulation. Drift occurs in populations that are not infinitely large. Allier, Moreau, *et al.* (2019) made a similar comparison for variances of 4-way crosses (see their Fig. 3) and reported higher correlations. This is likely because they simulated 50,000 progenies instead of 10,000 as we did, and because they considered fewer generations than we did. Both are expected to result in less drift. Thus, we do not deem our lower predictive correlations a reason for concern. This stochasticity is also the reason for the small apparent overestimation with the analytical method. We do not believe that overestimation is an actual effect of the proposed equations but rather caused by loss of variance due to drift in the simulated case. We chose a smaller number of individuals than Allier, Moreau, *et al.* (2019) for computational reasons. We included examples for the prediction of covariances and correlations between traits to illustrate that the developed equations do not only work for variances. Use cases may be scenarios in which breeders would like to change the correlation in the population toward a more favorable direction as discussed in literature (Abed and Belzile 2019; Neyhart *et al.* 2019; Wartha and Lorenz 2024).

Our approach may be understood as an extension of the equation presented by Allier, Moreau, *et al.* (2019) for calculating DH variances. In real-life breeding applications, breeders may be reluctant to create DH families based on crosses of higher orders than 4 because every higher order requires 1 more generation of crossing without selection. This increases the generation interval considered in the breeder's equation and thus decreases the genetic gain per unit time. That is why we do not expect large interest in the prediction of the DH variance itself but rather in the gametic MSV that can be calculated based on the DH variance. The creation of DHs is impossible in most livestock species, but we consider the DH variance just as a theoretical concept needed as a stepping stone to derive gametic MSV. The gametic MSV is a parameter that can be considered directly in selection in animal breeding. A few studies proposed to consider the gametic MSV of selection candidates (Santos *et al.* 2019; Wellmann and Bennewitz 2019; Bijma *et al.* 2020). Recently, we extended this idea by also considering the expected gametic MSV of offspring (Niehoff *et al.* 2024) to maximize genetic gain in the grandoffspring generation. This extends the planning horizon by 1 additional generation. Being able to calculate the gametic MSV of descendants in even more distant future generations enables to extend the planning horizon even further, which might bring benefits for breeding programs. We presented our idea of such a criterion for livestock breeding in Equation (1).

The equation for the *ExpBVSelGrGrGrOff* criterion is conceptually very similar to the one we presented in Niehoff *et al.* (2024) with the exception that this equation is simplified by being completely free of parameters describing the unknown mating partner from the population for individuals in the last generation. These parameters would be needed for animal breeding considerations because the “F1 ABCDEFGHxIJKLMNOP” individuals will be mated to selected animals of the population which in turn would require including terms that describe the variance of BVs of the population. Essentially, we avoid this by not assuming anything about the variance of gametic BVs produced by individuals of the population that are mated to the “F1 ABCDEFGHxIJKLMNOP” individuals. Instead, we simply take twice the genetic variance of gametes produced by the 16-way F1 $\left(2\sigma_{gametes\;F1\;ABCDEFGHxIJKLMNOP}^{2}\right)$ which approximates the variance of gametes produced by the individuals of the population that would be mated to individuals “F1 ABCDEFGHxIJKLMNOP” by assuming their gametic variance would be equally as high. This is in line with the approximation for the gametic MSV for the random mating partner of the population considered in the Index5 by Bijma *et al.* (2020). Bijma *et al.* (2020) used a first-order Taylor series to derive that the gametic MSV should be multiplied by 2. The result is however the same in that they multiply the variance of gametes by 2 to approximate the offspring variance. As a consequence, the criterion as presented here strictly describes the BV of selected F4 individuals that have the same selected F3 individuals as sires and dams. A worked out numerical example for the calculation of the *ExpBVSelGrGrGrOff* criterion can be found in Supplementary Material 1.

This *ExpBVSelGrGrGrOff* criterion may be used to select and position individuals in such a way in a mating plan that the BV in the great-great-grandoffspring generation is maximized. Please note that this is our idea of what a selection criterion looking more generations ahead may look like but other approaches could be possible too. Also, note that the proposed equation assumes that the distribution of BVs follows a normal distribution. Under directional selection and when the gametic MSV is approximately at least a quarter of the genetic variance in the previous generation, as in typical livestock breeding programs, the distribution of BVs will indeed be very close to normal. However, if this assumption is violated, e.g. because the gametic MSV of individuals is reduced a lot due to selfing, the BV distribution may deviate from normality in which case equations presented by Smith (2021) could be used to better model skewness and kurtosis.

Directional selection ultimately aims at increasing the frequency of beneficial alleles. In addition, selection also changes the linkage disequilibrium even in the infinitesimal model in which changes in allele frequency due to selection are assumed negligibly small (Walsh and Lynch 2018, chapters 16 and 24). However, for the analytical equations, it is assumed that no selection is happening, i.e. allele frequencies do not change and changes in linkage disequilibrium are only due to recombination but not due to selection. We included scenarios with selection in this study to investigate how much inaccuracy is caused by the obvious violation of the no-selection assumption in our gametic MSV prediction. We focus only on the gametic MSV as this is the most relevant metric and we believe we have sufficiently proven that we can predict all other properties well in the RANDOM scenario. The predictive ability for gametic MSV in the selected scenario was much lower than in the RANDOM scenarios, which emphasizes the clear deviation of real genomes with genes linked on chromosomes from the assumptions of the infinitesimal model. In addition, the prediction ability for gametic MSV in the selection scenario was much lower for corn (0.511) than for cattle (0.705), highlighting that prediction power is species dependent. Similarly, the gametic MSV for corn (27%) was more reduced due to selection than the one for cattle (21%). That the genome length, and thus the number of crossovers, has an effect on the reduction of the genetic variance of a population under directional selection was shown by Zhang and Hill (2005).

The simulation of the corn genome showed larger changes in allele frequency as well as more negative linkage disequilibrium than the cattle genome (Table 6). Thus, we conclude that the predictive ability of the gametic MSV with our derivation is higher for species with more chromosomes, or better, more effectively independent loci, since the assumptions of the infinitesimal model are likely met better. In practical applications, we would also expect that the predictive ability decreases more with higher selection intensities and the further ahead the generation for which variances are predicted. The higher predictive ability for larger genomes (cattle scenario) might also be counteracted by the lower accuracy of predicting SNP effects with genomic prediction models as e.g. Daetwyler's formula (Daetwyler *et al.* 2010) suggests lower prediction accuracy for species with larger genomes.

Note that what we call the “cattle” genome is just similar to cattle with regard to the number of chromosomes but not regarding chromosome length. Based on the genetic map published by Melzer *et al.* (2023), the average Holstein chromosome is about 0.83 Morgan long whereas the average corn chromosome, and thus also the average chromosome in our cattle genome scenario, is about 1.9 Morgan. We used three times the corn genome as a practical means of increasing the number of effectively independent loci 3-fold with the same trait architecture as in corn to better draw conclusions. Results for an actual cattle situation may deviate.

We have used the term “effectively independent” QTL in this study without much explanation of its definition so far. Our inspiration to consider the number of QTLs that are effectively independent as a theoretical concept rather than an actual number that can be measured stems from the publications of Zhong and Jannink (2007) and Wright (1968). In chapter 15, Wright (1968) introduces the concept of “independent loci” as the number of unlinked QTL of equal and completely additive effect that would produce the same additive genetic variance in F2 as observed in actual F2 individuals obtained from selfing F1 individuals of a cross between two homozygous lines. This definition is in line with our use of the term “effectively independent QTL” in this study.

Since variances are different in case individuals are selected, one might think that we should aim to incorporate the effects of selection in the analytical equations. Although it is a good idea, it is unfortunately challenging for two reasons. First, we would need to be able to predict the allele frequency change in expected selected descendant individuals. Second, and this is likely the more challenging issue, the alleles in selected individuals are not independent from each other even when accounting for linkage and meiosis. This is because in a selected individual, the presence of a beneficial allele at a locus makes the presence of another beneficial allele at the same or a different locus less likely, even if the two QTLs are on different chromosomes. Mathematically speaking, selection induces a negative covariance between QTLs (Bulmer 1971). The reader interested in incorporating the effect of selection is referred to chapters 5 and 8 of Walsh and Lynch (2018) as a starting point.

A straightforward approach to considering selection would be to stochastically simulate the mating scheme as done in this study. The disadvantage of stochastic simulation is that it takes considerably more time than our calculations. For comparison, evaluating one 16-way cross took a couple of seconds whereas the simulation approach with MoBPS took some minutes. Since the genetic level after 4 generations of selection could be predicted with high accuracy, despite not modeling the effect of selection (see Table 7), a potential hybrid approach could be employed. This approach would involve evaluating all mating plans analytically and then reevaluating only the e.g. top 5% by means of simulations.

It may also be that the imprecision of QTL effect estimation methods renders all efforts to model allele frequency and linkage disequilibrium change meaningless. Even the prediction of the next-generation genetic variance, which cannot be influenced by selection, may be unsatisfactorily inaccurate in some real breeding programs (Rembe *et al.* 2022; Danguy des Déserts *et al.* 2023). Further studies would be needed for evaluation.

## Conclusion

We developed analytical equations that enable to predict the genetic variance based on any number of founding haplotypes. This variance may be decomposed into gametic MSVs of ancestors which may then be used in a selection criterion. The comparison of the algebraic prediction obtained with our method with results from simulation validated that our equation is estimating variances properly. The prediction accuracy for variances of selected individuals was much lower. The prediction accuracy of the genetic level of individuals in 5 generations was only mildly influenced by the inaccurate variance prediction. Given the faster prediction with the analytical approach compared to simulation, the inaccuracy may be acceptable. Further testing is needed to evaluate the effect if input parameters are estimated.

## Supplementary Material

jkae205_Supplementary_Data

## Data Availability

The MoBPS simulation and R functions to calculate variances are provided in Supplementary Material 2 of this paper. The genotypes and genetic map used as input are available in Supplementary File S4 of Allier, Moreau, *et al.* (2019). An option to test the functionality without the inputs from Allier, Moreau, *et al.* (2019) is provided in the comments in R script. The MoBPS version 1.11.40 that was used for this study can be downloaded from Torsten Pook's GitHub repository (https://github.com/tpook92/MoBPS). Supplemental material available at G3 online.

## Appendix

### Example for 4 times higher DH MSV than gametic MSV

This example will show the calculation of the gametic MSV and the variance of DH progeny developed from a single F1 individual. Suppose only 2 QTLs are affecting a trait. The beneficial alleles in this example will be indicated by a capital letter (A, B), and unfavorable alleles will be indicated with a lowercase letter (a, b). Suppose the A allele has an additive effect of 10 units and the B allele of 1 unit. The unfavorable alleles have 0 effect. Suppose an individual is heterozygous at both of these loci. Further, suppose the beneficial alleles are linked in coupling phase, i.e. are on the same chromosome. Assume the genetic distance between the A and B loci is 0.26 Morgan. The expected recombination frequency can be calculated based on Haldane's mapping function (Haldane 1919) with $rf=0.5*(1-e^{-2*M})$ with *M* as the genetic distance in units Morgan. According to the genetic distance, we expect a recombination frequency of 20%, meaning the haplotypes as observed in the individual will be broken in 20 out of 100 meiotic events. The expected variance of BVs among gametes produced by this individual is 28.25 whereas the expected variance of BVs among DH lines produced from this individual is 113, so 4 times larger. The calculations are shown in Tables A1 and A2.

**Table A1.** Calculation of gametic MSV of the example considering allele substitution effects and recombination.

Gamete genotypes	Expected frequency gametes	Gametic breeding value (BV)	Frequency × (BV − average BV)^2^
AB	0.4	11	12.1
Ab	0.1	10	2.025
aB	0.1	1	2.025
ab	0.4	0	12.1
		Average BV: 5.5	Sum (variance of BV): 28.25

**Table A2.** Calculation of DH MSV of the example considering allele substitution effects and recombination.

DH genotypes	Expected frequency DHs	DH breeding value (BV)	Frequency × (BV − average BV)^2^
AABB	0.4	22	48.1
AAbb	0.1	20	8.1
aaBB	0.1	2	8.1
aabb	0.4	0	48.1
		Average BV: 11	Sum (variance of BV): 113

## References

[jkae205-B1] Abed A , Belzile F. 2019. Exploring the realm of possibilities: trying to predict promising crosses and successful offspring through genomic mating in barley. Crop Breed Genet Genomics. 1:e190019. doi:10.20900/cbgg20190019.

[jkae205-B2] Allier A , Lehermeier C, Charcosset A, Moreau L, Teyssèdre S. 2019. Improving short- and long-term genetic gain by accounting for within-family variance in optimal cross-selection. Front Genet. 10:1006. doi:10.3389/fgene.2019.01006. 31737033 PMC6828944

[jkae205-B3] Allier A , Moreau L, Charcosset A, Teyssèdre S, Lehermeier C. 2019. Usefulness criterion and post-selection parental contributions in multi-parental crosses: application to polygenic trait introgression. G3 (Bethesda). 9(5):1469–1479. doi:10.1534/g3.119.400129. 30819823 PMC6505154

[jkae205-B4] Bijma P , Wientjes YCJ, Calus MPL. 2020. Breeding top genotypes and accelerating response to recurrent selection by selecting parents with greater gametic variance. Genetics. 214(1):91–107. doi:10.1534/genetics.119.302643. 31772074 PMC6944402

[jkae205-B5] Bonk S , Reichelt M, Teuscher F, Segelke D, Reinsch N. 2016. Mendelian sampling covariability of marker effects and genetic values. Genet Sel Evol. 48:36. doi:10.1186/s12711-016-0214-0. 27107720 PMC4842306

[jkae205-B6] Bulmer MG . 1971. The effect of selection on genetic variability. Am Nat. 105(943):201–211. doi:10.1086/282718.

[jkae205-B7] Daetwyler HD , Pong-Wong R, Villanueva B, Woolliams JA. 2010. The impact of genetic architecture on genome-wide evaluation methods. Genetics. 185(3):1021–1031. doi:10.1534/genetics.110.116855. 20407128 PMC2907189

[jkae205-B8] Danguy des Déserts A , Durand N, Servin B, Goudemand-Dugué E, Alliot JM, Ruiz D, Charmet G, Elsen JM, Bouchet S. 2023. Comparison of genomic-enabled cross selection criteria for the improvement of inbred line breeding populations. G3 (Bethesda). 13(11):jkad195. doi:10.1093/g3journal/jkad195. 37625792 PMC10627264

[jkae205-B9] de Abreu Santos DJ , Cole JB, Liu GE, VanRaden PM, Ma L. 2020. Gamevar.f90: a software package for calculating individual gametic diversity. BMC Bioinformatics. 21(1):100. doi:10.1186/s12859-020-3417-x. 32143564 PMC7060627

[jkae205-B10] Dempfle L . 1990. Problems in the use of the relationship matrix in animal breeding. In: Gianola D, Hammond K, editors. Advances in Statistical Methods for Genetic Improvement of Livestock. Berlin (Heidelberg): Springer Berlin Heidelberg. p. 454–473.

[jkae205-B11] Falconer DS , Mackay TFC. 1996. Introduction to Quantitative Genetics. 4th ed. Harlow: Longman Group Ltd.

[jkae205-B12] Haldane JBS . 1919. The probable errors of calculated linkage values, and the most accurate method of determining gametic from certain zygotic series. J Genet. 8(4):291–297. doi:10.1007/BF02983270.

[jkae205-B13] Hill WG , Weir BS. 2011. Variation in actual relationship as a consequence of Mendelian sampling and linkage. Genet Res (Camb). 93(1):47–64. doi:10.1017/S0016672310000480. 21226974 PMC3070763

[jkae205-B14] Lehermeier C , Teyssèdre S, Schön CC. 2017. Genetic gain increases by applying the usefulness criterion with improved variance prediction in selection of crosses. Genetics. 207(4):1651–1661. doi:10.1534/genetics.117.300403. 29038144 PMC5714471

[jkae205-B15] Melzer N , Qanbari S, Ding X, Wittenburg D. 2023. CLARITY: a Shiny app for interactive visualisation of the bovine physical-genetic map. Front Genet. 14:1082782. doi:10.3389/fgene.2023.1082782. 37323679 PMC10267868

[jkae205-B16] Meuwissen TH . 1997. Maximizing the response of selection with a predefined rate of inbreeding. J Anim Sci. 75(4):934–940. doi:10.2527/1997.754934x. 9110204

[jkae205-B17] Meuwissen THE , Sonesson AK, Gebregiwergis G, Woolliams JA. 2020. Management of genetic diversity in the era of genomics. Front Genet. 11:880. doi:10.3389/fgene.2020.00880. 32903415 PMC7438563

[jkae205-B18] Mohammadi M , Tiede T, Smith KP. 2015. PopVar: a genome-wide procedure for predicting genetic variance and correlated response in biparental breeding populations. Crop Sci. 55(5):2068–2077. doi:10.2135/cropsci2015.01.0030.

[jkae205-B19] Musa AA , Reinsch N. 2023. A similarity matrix for preserving haplotype diversity among parents in genomic selection. bioRxiv 543227. 10.1101/2023.06.01.543227, preprint: not peer reviewed. PMC12501756 40035336

[jkae205-B20] Neyhart JL , Lorenz AJ, Smith KP. 2019. Multi-trait improvement by predicting genetic correlations in breeding crosses. G3 (Bethesda). 9(10):3153–3165. doi:10.1534/g3.119.400406. 31358561 PMC6778794

[jkae205-B21] Niehoff TAM , Ten Napel J, Bijma P, Pook T, Wientjes YCJ, Hegedűs B, Calus MPL. 2024. Improving selection decisions with mating information by accounting for Mendelian sampling variances looking two generations ahead. Genet Sel Evol. 56(1):41. doi:10.1186/s12711-024-00899-2. 38773363 PMC11107025

[jkae205-B22] Osthushenrich T , Frisch M, Herzog E. 2017. Genomic selection of crossing partners on basis of the expected mean and variance of their derived lines. PLoS One. 12(12):e0188839. doi:10.1371/journal.pone.0188839. 29200436 PMC5714341

[jkae205-B23] Pook T , Schlather M, Simianer H. 2020. MoBPS—modular breeding program simulator. G3 (Bethesda). 10(6):1915–1918. doi:10.1534/g3.120.401193. 32229505 PMC7263682

[jkae205-B24] Rembe M , Zhao Y, Wendler N, Oldach K, Korzun V, Reif JC. 2022. The potential of genome-wide prediction to support parental selection, evaluated with data from a commercial barley breeding program. Plants (Basel). 11(19):2564. doi:10.3390/plants11192564. 36235430 PMC9571379

[jkae205-B25] Santos DJA , Cole JB, Lawlor TJ Jr, VanRaden PM, Tonhati H, Ma L. 2019. Variance of gametic diversity and its application in selection programs. J Dairy Sci. 102(6):5279–5294. doi:10.3168/jds.2018-15971. 30981488

[jkae205-B26] Schnell F , Utz H. 1975. Bericht über die Arbeitstagung der Vereinigung österreichischer Pflanzenzüchter. Gumpenstein, Austria: BAL Gumpenstein. p. 243–248.

[jkae205-B27] Scott MF , Ladejobi O, Amer S, Bentley AR, Biernaskie J, Boden SA, Clark M, Dell’Acqua M, Dixon LE, Filippi CV, et al 2020. Multi-parent populations in crops: a toolbox integrating genomics and genetic mapping with breeding. Heredity (Edinb). 125(6):396–416. doi:10.1038/s41437-020-0336-6. 32616877 PMC7784848

[jkae205-B28] Segelke D , Reinhardt F, Liu Z, Thaller G. 2014. Prediction of expected genetic variation within groups of offspring for innovative mating schemes. Genet Sel Evol. 46(1):42. doi:10.1186/1297-9686-46-42. 24990472 PMC4118311

[jkae205-B29] Smith RD , 2021 Quantitative traits under selection: derivations of distributions, higher moments, and the effects of recombination. bioRxiv: 454547. 10.1101/2021.07.30.454547, preprint: not peer reviewed

[jkae205-B30] Walsh B , Lynch M. 2018. Evolution and Selection of Quantitative Traits. Oxford: Oxford University Press.

[jkae205-B31] Wartha CA , Lorenz AJ. 2024. Genomic predictions of genetic variances and correlations among traits for breeding crosses in soybean. Heredity (Edinb). 133(3):173–185 doi:10.1038/s41437-024-00703-3. 38997517 PMC11350137

[jkae205-B32] Wellmann R , Bennewitz J. 2019. Key genetic parameters for population management. Front Genet. 10:667. doi:10.3389/fgene.2019.00667. 31475027 PMC6707806

[jkae205-B33] Wolfe MD , Chan AW, Kulakow P, Rabbi I, Jannink JL. 2021. Genomic mating in outbred species: predicting cross usefulness with additive and total genetic covariance matrices. Genetics. 219(3):iyab122. doi:10.1093/genetics/iyab122. 34740244 PMC8570794

[jkae205-B34] Woolliams JA , Berg P, Dagnachew BS, Meuwissen TH. 2015. Genetic contributions and their optimization. J Anim Breed Genet. 132(2):89–99. doi:10.1111/jbg.12148. 25823835

[jkae205-B35] Wright S . 1968. Evolution and the Genetics of Populations: A Treatise in Four Volumes. Genetic and Biometric Foundations. Vol. 1. Chicago: Univeristy of Chicago Press.

[jkae205-B36] Zhang XS , Hill WG. 2005. Predictions of patterns of response to artificial selection in lines derived from natural populations. Genetics. 169(1):411–425. doi:10.1534/genetics.104.032573. 15677752 PMC1448869

[jkae205-B37] Zhong S , Jannink JL. 2007. Using quantitative trait loci results to discriminate among crosses on the basis of their progeny mean and variance. Genetics. 177(1):567–576. doi:10.1534/genetics.107.075358. 17660556 PMC2013701

