# Supplementary material for: Prediction of additive genetic variances of descendants for complex families based on Mendelian sampling variances
Source: G3 (Bethesda). 2024 Aug 28;14(11):jkae205. doi: 10.1093/g3journal/jkae205 (PMC11540313; doi:10.1093/g3journal/jkae205)
Supplement: jkae205_Supplementary_Data [file jkae205_supplementary_data.zip › Supplemental_Material_S1_G3-2024-405313.docx]

Supplementary Material S1

Numerical example for inferring the gametic MSV based on predicted DH variances

Assume the 16-way crossing scheme as shown in Table 3 is of interest. Suppose the variances of all DH groups are calculated. Their values are shown below:

$\sigma_{2-way DH AB}^{2}$=0.94

$\sigma_{2-way DH CD}^{2}$=1.02

$\sigma_{2-way DH EF}^{2}$=0.92

$\sigma_{2-way DH GH}^{2}$=1.16

$\sigma_{2-way DH IJ}^{2}$=1.03

$\sigma_{2-way DH KL}^{2}$=0.92

$\sigma_{2-way DH MN}^{2}$=1.05

$\sigma_{2-way DH OP}^{2}$=1.07

$\sigma_{4-way DH ABCD}^{2}$=1.56

$\sigma_{4-way DH EFGH}^{2}$=1.47

$\sigma_{4-way DH IJKL}^{2}$=1.65

$\sigma_{4-way DH MNOP}^{2}$=1.54

$\sigma_{8-way DH ABCDEFGH}^{2}$=1.69

$\sigma_{8-way DH IJKLMNOP}^{2}$=1.53

$\sigma_{16-way DH ABCDEFGHIJKLMNOP}^{2}$=1.99

The gametic MSVs of the 2-way F1s can be calculated by taking the DH variance and dividing by 4:

$\sigma_{2-way gamMS AxB}^{2}=\frac{\sigma_{2-way DH AB}^{2}}{4}$=0.94/4=0.2350

$\sigma_{2-way gamMS CxD}^{2}$=1.02/4=0.2550

$\sigma_{2-way gamMS ExF}^{2}$=0.92/4=0.2300

$\sigma_{2-way gamMS GxH}^{2}$=1.16/4=0.2900

$\sigma_{2-way gamMS IxJ}^{2}$=1.03/4=0.2575

$\sigma_{2-way gamMS KxL}^{2}$=0.92/4=0.2300

$\sigma_{2-way gamMS MxN}^{2}$=1.05/4=0.2625

$\sigma_{2-way gamMS OxP}^{2}$=1.07/4=0.2675

The variance of 4-way F1 individuals can be calculated based on the 2-way F1 gametic MSVs:

$\sigma_{4-way F1 ABxCD}^{2}$=$\sigma_{2-way gamMS AxB}^{2}+\sigma_{2-way gamMS CxD}^{2}$=0.2350+0.2550=0.49

$\sigma_{4-way F1 EFxGH}^{2}$=0.2300+0.2900=0.52

$\sigma_{4-way F1 IJxKL}^{2}$=0.2575+0.2300=0.4875

$\sigma_{4-way F1 MNxOP}^{2}$=0.2625+0.2675=0.53

The gametic MSVs of 4-way F1s can be calculated based on the 4-way F1 variance and the 4-way DH variance with:

$\sigma_{4-way gamMS ABxCD}^{2}$=$\left( \frac{\sigma_{4-way DH ABCD}^{2}-\sigma_{4-way F1 ABxCD}^{2}}{4} \right)$=(1.56 – 0.49) /4=0.2675

$\sigma_{4-way gamMS EFxGH}^{2}$=(1.47 – 0.52) /4=0.2375

$\sigma_{4-way gamMS IJxKL}^{2}$=(1.65 – 0.4875) /4=0.2906

$\sigma_{4-way gamMS MNxOP}^{2}$=(1.54 – 0.53) /4=0.2525

The variance of 8-way F1 individuals can be calculated based on the 4-way F1 variances and the 4-way F1 gametic MSVs:

$$\sigma_{8-way F1 ABCDxEFGH}^{2}$$

$$=\frac{\sigma_{4-way F1 ABxCD}^{2}}{4}+\frac{\sigma_{4-way F1 EFxGH}^{2}}{4}+\sigma_{4-way gamMS ABxCD}^{2}+\sigma_{4-way gamMS EFxGH}^{2}$$

$=$0.49/4 + 0.52/4 + 0.2675 + 0.2375 = 0.7575

$\sigma_{8-way F1 IJKLxMNOP}^{2}=$0.4875/4 + 0.53/4 + 0.2906 + 0.2525 = 0.7975

The gametic MSVs of 8-way F1s can be calculated based on the 8-way F1 variance and the 8-way DH variance with:

$\sigma_{8-way gamMS ABCDxEFGH}^{2}=\left( \frac{\sigma_{8-way DH ABCDEFGH}^{2}-\sigma_{8-way F1 ABCDxEFGH}^{2}}{4} \right)=$(1.69 – 0.7575)/4 = 0.2331

$\sigma_{8-way gamMS IJKLxMNOP}^{2}=$(1.53 – 0.7975)/4 = 0.1831

The variance of 16-way F1 individuals can be calculated based on the 8-way F1 variances and the 8-way F1 gametic MSVs:

$$\sigma_{16-way F1 ABCDEFGHxIJKLMNOP}^{2}$$

$$=\frac{\sigma_{8-way F1 ABCDxEFGH}^{2}}{4}+\frac{\sigma_{8-way F1 IJKLxMNOP}^{2}}{4}+\sigma_{8-way gamMS ABCDxEFGH}^{2}+\sigma_{8-way gamMS IJKLxMNOP}^{2}$$

= 0.7575/4 + 0.7975/4 + 0.2331 + 0.1831 = 0.805

The gametic MSVs of 16-way F1s can be calculated based on the 16-way F1 variance and the 16-way DH variance with:

$$\sigma_{16-way gamMS ABCDEFGHxIJKLMNOP}^{2}$$

$$=\left( \frac{\sigma_{16-way DH ABCDEFGHIJKLMNOP}^{2}-\sigma_{16-way F1 ABCDEFGHxIJKLMNOP}^{2}}{4} \right)$$

=(1.99 – 0.805) /4

=0.29625

Exemplary calculation of the proposed selection criterion looking 5 generations ahead:

Here, the same values as used above will be used. For simplicity, we assume that the selection intensities that are to be applied in any future generation are the same, i.e., $i_{F1}=i_{F2}=i_{F3}=i_{F4}=i$. We assume that the top 5% of the population are selected which corresponds to $i=2.06$. With this selection intensity, the variance reduction coefficient $k$ is 0.86. $k$ is a function of the selection intensity and calculated as $k=i*(i-x)$ with $i$ being the selection intensity and $x$ being the normalized truncation selection point. The R script in Supplementary Material S2 contains the function ‘ixk()’ which calculates the properties based on a supplied selected fraction. This example represents an animal breeding case, meaning the founders are at least partially heterozygous. The breeding values of the 8 founders are:

${BV}_{AB}$=-0.04

${BV}_{CD}$=-0.02

${BV}_{EF}$=0.94

${BV}_{GH}$=0.82

${BV}_{IJ}$=0.59

${BV}_{KL}$=0.92

${BV}_{MN}$=0.78

${BV}_{OP}$=0.07

The 4-way F1 variances are the same as above. For all variances of later generations, the fact that previous generations have been selected need to be considered. In the following, we describe how to calculate the variance based on gametic MSV derived in the section above and the variances of selected parents.

$\sigma_{selected 4-way F1 ABxCD}^{2}=\left( 1-k \right)*{\sigma^{2}}_{F1 ABxCD}$=(1-0.86)*0.49=0.0677

$\sigma_{selected 4-way F1 EFxGH}^{2}$=(1-0.86)*0.52=0.0718

$\sigma_{selected 4-way F1 IJxKL}^{2}$=(1-0.86)*0.4875=0.0673

$\sigma_{selected 4-way F1 MNxOP}^{2}$=(1-0.86)*0.53=0.0732

Based on the variance among the selected 4-way F1 parents and the gametic MSV of 4-way F1 parents, the variance of the unselected 8-way F1 individuals can be derived. Note that we assume that the gametic MSV calculated in the above section for a random individual is representative for the gametic MSV of a selected individual.

$$\sigma_{8-way F1 ABCDxEFGH from selected parents}^{2}$$

$$=\left( \frac{\sigma_{selected 4-way F1 ABxCD}^{2}}{4}+\frac{\sigma_{selected 4-way F1 EFxGH}^{2}}{4}+\sigma_{4-way gamMS ABxCD}^{2}+\sigma_{4-way gamMS EFxGH}^{2} \right)$$

$=$(0.0677/4 + 0.0718/4 + 0.2675 + 0.2375)
=0.5399

$$\sigma_{8-way F1 IJKLxMNOP from selected parents}^{2}$$

$$=\left( \frac{\sigma_{selected 4-way F1 IJxKL}^{2}}{4}+\frac{\sigma_{selected 4-way F1 MNxOP}^{2}}{4}+\sigma_{4-way gamMS IJxKL}^{2}+\sigma_{4-way gamMS MNxOP}^{2} \right)$$

=(0.0673/4 + 0.0732/4 + 0.2906 + 0.2525)
=0.5782

Next, the variance of the selected 8-way F1 individuals can be calculated. It is needed for the calculation of 16-way F1 variances.

$\sigma_{selected 8-way F1 ABCDxEFGH}^{2}=\left( 1-k \right)$*$\sigma_{8-way F1 ABCDxEFGH from selected parents}^{2}$
=(1-0.86)*0.5399
=0.0745

$\sigma_{selected 8-way F1 IJKLxMNOP}^{2}=\left( 1-k \right)$*$\sigma_{8-way F1 IJKLxMNOP from selected parents}^{2}$
=(1-0.86)*0.5782
=0.0798

$$\sigma_{16-way F1 ABCDEFGHxIJKLMNOP from selected parents}^{2}$$

$$=\left( \begin{aligned} \frac{\sigma_{selected 8-way F1 ABCDxEFGH}^{2}}{4}+\frac{\sigma_{selected 8-way F1 IJKLxMNOP}^{2}}{4} \\ +\sigma_{8-way gamMS ABCDxEFGH}^{2}+\sigma_{8-way gamMS IJKLxMNOP}^{2} \end{aligned} \right)$$

=(0.0745/4 + 0.0798/4 + 0.2331 + 0.1831)
=0.4548

The individuals of the last generation ($\sigma_{32-way F1 ABCDEFGHIJKLMNOPxNA}^{2}$) can be traced back to 16 great-great-grandparents, or 32 haplotypes. However, in the example here, we only consider 16 haplotypes. The variance of the 32-way F1 individuals is the sum of the variance of gametes of the paternal selection path and the maternal selection path. The variance of one of these paths can be calculated (regarded as the “paternal path” here). The variance among breeding values of the 32-way F1s can be approximated by assuming that the variance of gametes of the paternal path is identical to the variance of the maternal path.

$$\sigma_{selected 16-way F1 ABCDEFGHxIJKLMNOP}^{2}$$

$$=\left( 1-k \right)\sigma_{16-way F1 ABCDEFGHxIJKLMNOP from selected parents}^{2}$$

=(1-0.86)*0.4548
=0.0628

$$\sigma_{gametes 16-way F1 ABCDEFGHxIJKLMNOP \left( paternal \right)}^{2}$$

$$=\frac{\sigma_{selected 16-way F1 ABCDEFGHxIJKLMNOP}^{2}}{4}+\sigma_{16-way gamMS ABCDEFGHxIJKLMNOP}^{2}$$

=0.0628/4 + 0.29625
=0.312

$${approx. \sigma}_{32-way F1 ABCDEFGHIJKLMNOPxNA}^{2}$$

$$=\sigma_{gametes 16-way F1 ABCDEFGHxIJKLMNOP \left( paternal \right)}^{2}+\sigma_{gametes 16-way F1 NA \left( maternal \right)}^{2}$$

$$=2*\sigma_{gametes 16-way F1 ABCDEFGHxIJKLMNOP \left( paternal \right)}^{2}$$

=2*0.312
=0.6239

All necessary variances are calculated now and can be filled into the proposed selection criterion:

$$ExpBVSelGrGrGrOff$$

$$= \frac{{BV}_{AB}+{BV}_{CD}+{BV}_{EF}+{BV}_{GH}+{BV}_{IJ}+{BV}_{KL}+{BV}_{MN}+{BV}_{OP}}{8}+$$

$$i_{F1}\left( \frac{\sigma_{F1 ABxCD}+\sigma_{F1 EFxGH}+\sigma_{F1 IJxKL}+\sigma_{F1 MNxOP}}{4} \right)+$$

$$i_{F2}\left( \frac{\sigma_{F1 ABCDxEFGH}+\sigma_{F1 IJKLxMNOP}}{2} \right)+$$

$$i_{F3}\sigma_{F1 ABCDEFGHxIJKLMNOP}+$$

$$i_{F4}\sqrt{2\sigma_{gametes F1 ABCDEFGHxIJKLMNOP}^{2}}$$

$$=\frac{\left( -0.04 \right)+\left( -0.02 \right)+0.94+0.82+0.59+0.92+0.78+0.07}{8}+$$

$$2.06\left( \frac{\sqrt{0.49}+\sqrt{0.52}+\sqrt{0.4875}+\sqrt{0.53}}{4} \right)+$$

$$2.06\left( \frac{\sqrt{0.5399}+\sqrt{0.5782}}{2} \right)+$$

$$2.06\sqrt{0.4548}+$$

$$2.06\sqrt{0.6239}$$

=4.9314
