# Supplementary material for: Prediction of additive genetic variances of descendants for complex families based on Mendelian sampling variances
Source: G3 (Bethesda). 2024 Aug 28;14(11):jkae205. doi: 10.1093/g3journal/jkae205 (PMC11540313; doi:10.1093/g3journal/jkae205)
Supplement: jkae205_Supplementary_Data [file jkae205_supplementary_data.zip › Supplemental_Material_S3_G3-2024-405313.docx]

Supplementary Material S3

Visualization of the observed genetic level of selected 16-way F2 individuals versus the parent average breeding values, and the genetic level predicted with the ExpBVSelGrGrGrOff criterion when using the proposed equation for variance prediction, respectively.

| **Corn** | **Cattle** |
| --- | --- |
| 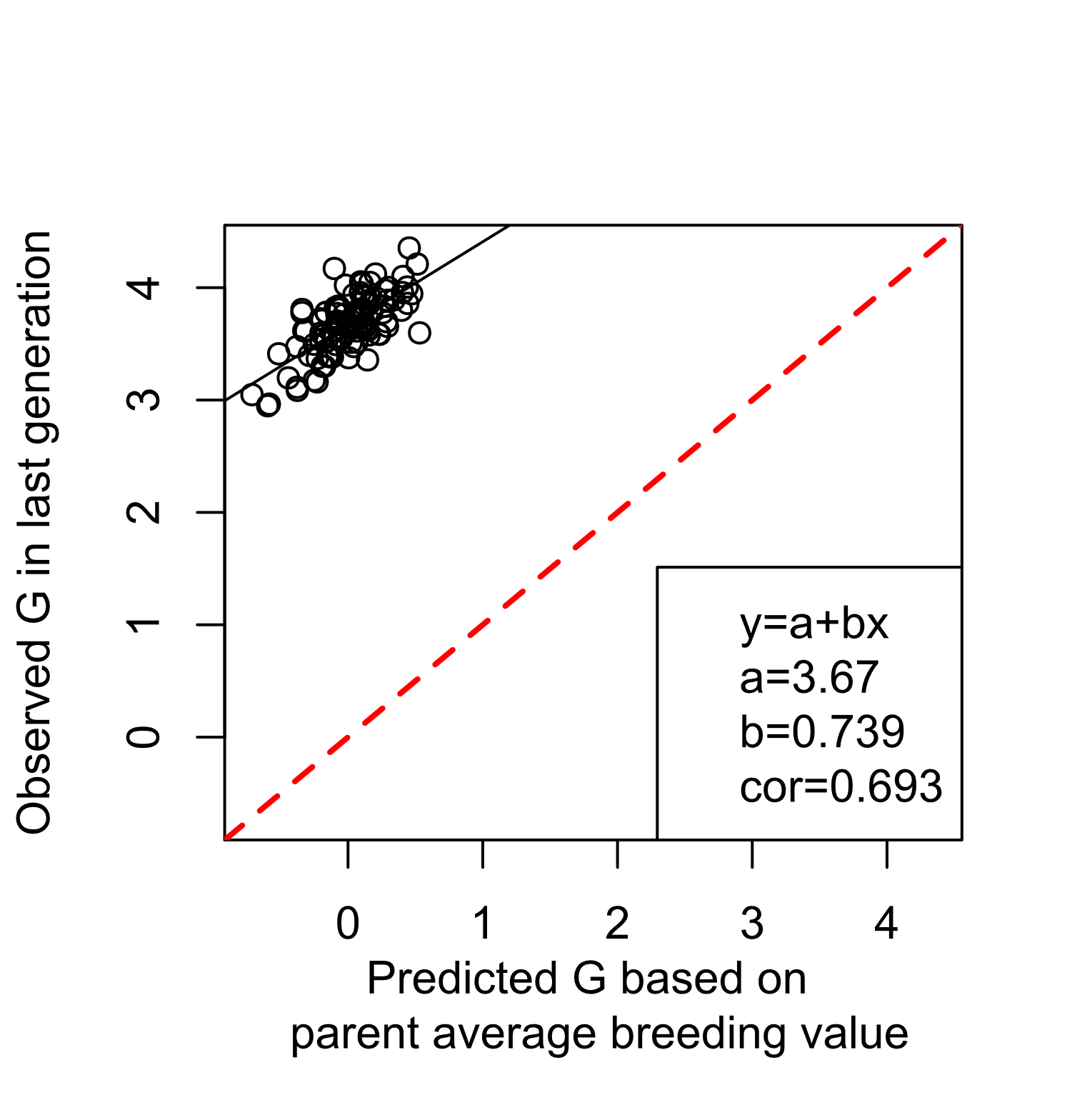 | 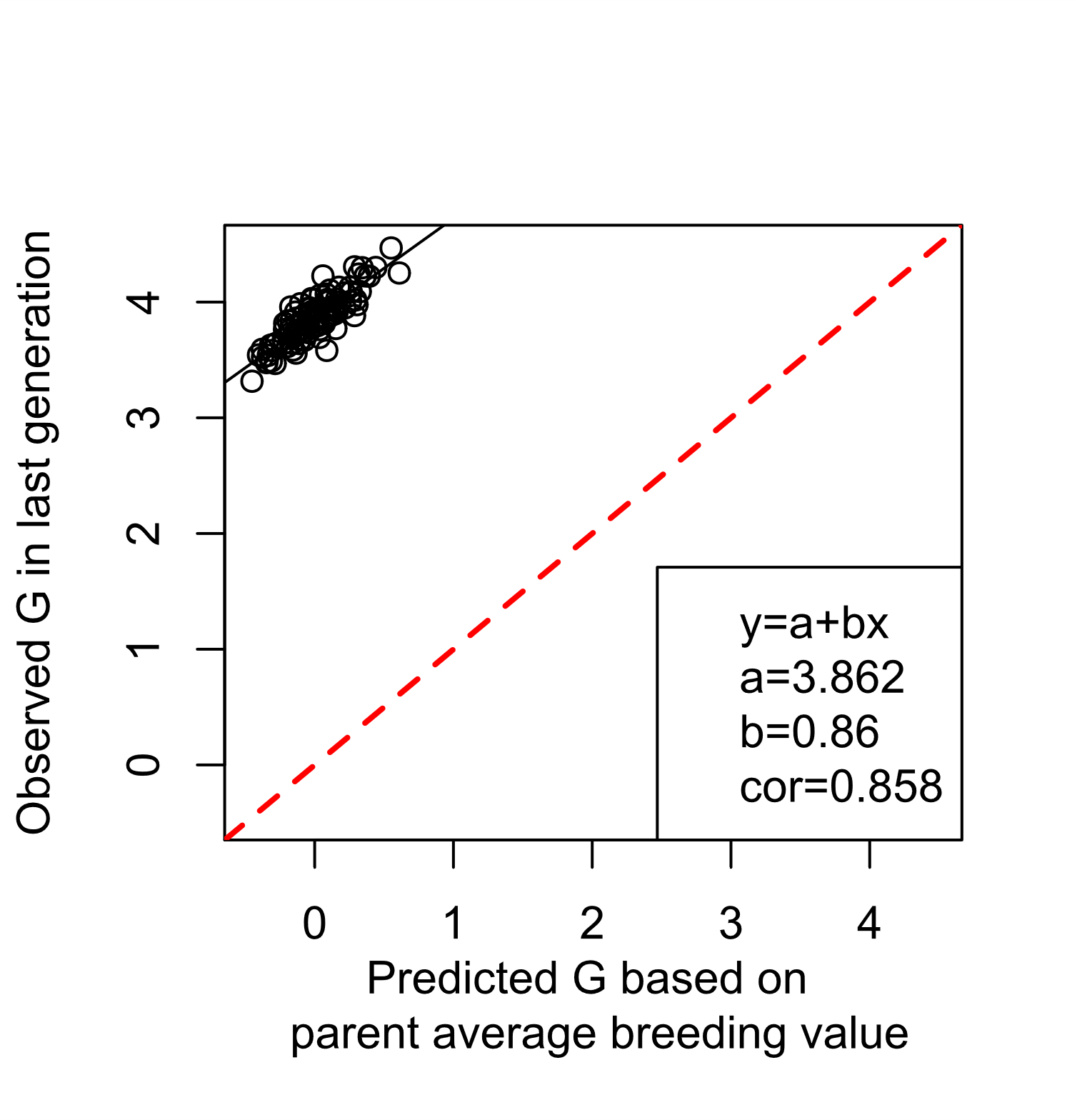 |
| 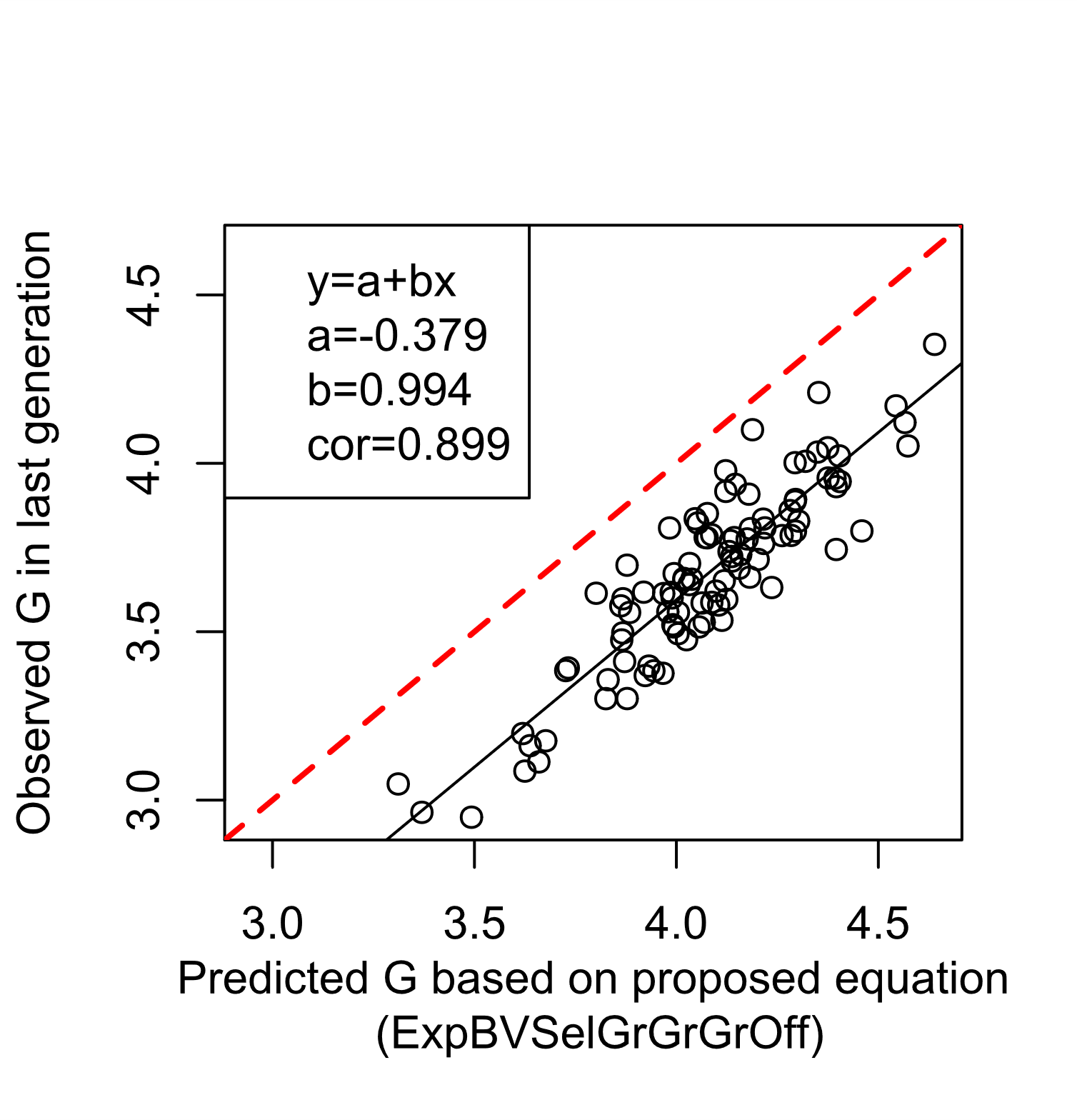 | 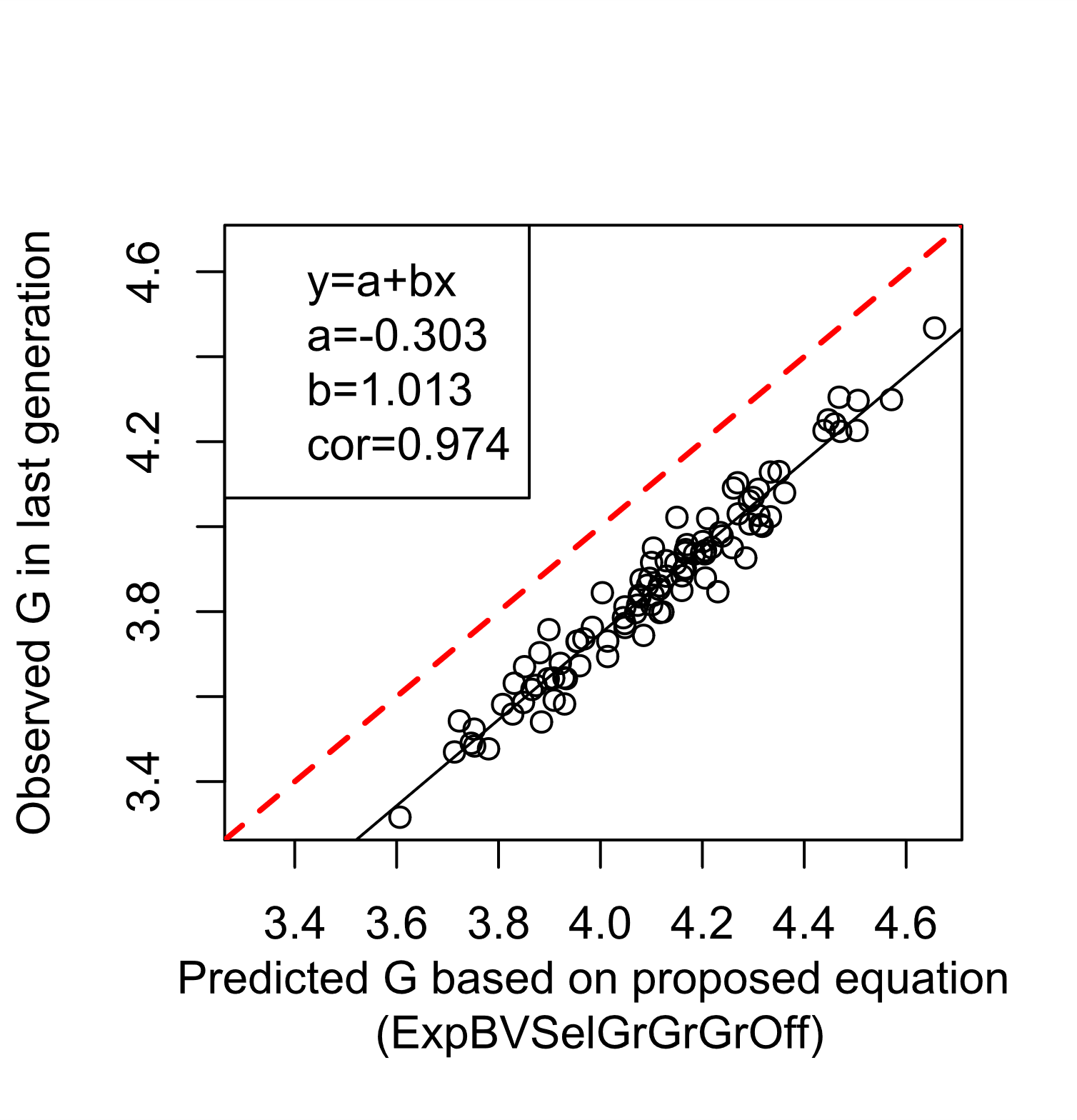 |
